# Natural history of depression up to 18 years after stroke: a population-based South London Stroke Register study

**DOI:** 10.1016/j.lanepe.2024.100882

**Published:** 2024-03-25

**Authors:** Lu Liu, Iain J. Marshall, Ruonan Pei, Ajay Bhalla, Charles DA. Wolfe, Matthew DL. O’Connell, Yanzhong Wang

**Affiliations:** a School of Life Course and Population Sciences, King's College London, London, United Kingdom; b NIHR Applied Research Collaboration (ARC) South London, London, United Kingdom; c Department of Ageing Health and Stroke, Guy’s and St Thomas’ National Health Service Foundation Trust and King’s College London, United Kingdom

**Keywords:** Stroke, Depression, Psychiatric disorders, Natural history

## Abstract

**Background:**

Current evidence on the long-term natural history of post-stroke depression (PSD) is limited. We aim to determine the prevalence, incidence, duration and recurrence rates of depression to 18-years after stroke and assess differences by onset-time and depression severity.

**Methods:**

Data were from the South London Stroke Register (1995–2019, N = 6641 at registration). Depression was defined using the Hospital Anxiety and Depression scale (scores > 7 = depression) at 3-months, then annually to 18-years after stroke. We compared early- (3-months post-stroke) vs late-onset depression (1-year) and initial mild (HADS scores > 7) vs severe depression (scores > 10).

**Findings:**

3864 patients were assessed for depression at any time-points during the follow-up (male:55.4% (2141), median age: 68.0 (20.4)), with the number ranging from 2293 at 1-year to 145 at 18-years after stroke. Prevalence of PSD ranged from 31.3% (28.9–33.8) to 41.5% (33.6–49.3). The cumulative incidence of depression was 59.4% (95% CI 57.8–60.9), of which 87.9% (86.5–89.2) occurred within 5-years after stroke. Of patients with incident PSD at 3-months after stroke, 46.6% (42.1–51.2) recovered after 1 year. Among those recovered, 66.7% (58.0–74.5) experienced recurrent depression and 94.4% (87.5–98.2) of recurrences occurred within 5-years since recovery. Similar estimates were observed in patients with PSD at 1-year. 34.3% (27.9–41.1) of patients with severe depression had recovered at the next time-point, compared to 56.7% (50.5–62.8) with mild depression. Recurrence rate at 1-year after recovery was higher in patients with severe depression (52.9% (35.1–70.2)) compared to mild depression (23.5% (14.1–35.4)) (difference: 29.4% (7.6–51.2), p = 0.003).

**Interpretation:**

Long-term depressive status may be established by 5-years post-onset. Early- and late-onset depression presented similar natural history, while severe depression had a longer duration and quicker recurrence than mild depression. These estimates were limited to alive patients completing the depression assessment, who tended to have less severe stroke than excluded patients, so may be underestimated and not generalizable to all stroke survivors.

**Funding:**

10.13039/501100000272National Institute for Health and Care Research (NIHR202339).

**Research in context**

**Evidence before this study**

Studies published up to 4 November 2022 on Medline, Embase, PsycINFO, and Web of Science Core Collection were searched. We used terms such as “stroke”, “post-stroke”, “cerebrovascular diseases”, “intracerebral haemorrhage”, “ischemic”, “depression”, “depressive disorder”, “prevalence”, “frequency”, “incidence”, “frequency” and “natural history” looking for matches in publications. There were no restrictions on the basis of language, sample size or duration of follow-up. We published a systematic review and meta-analysis on the natural history of post-stroke depression (PSD) which included 24 studies and 13 studies (out of 24 studies) showed low risk of bias. The pooled estimates showed that stroke survivors with early-onset depression (within 3 months after stroke) were at high risks for remaining depressed and make up two thirds of the incident cases during 1 year after stroke. However, there is only one study with follow-ups beyond 5-years after stroke. Therefore, there is a lack of evidence on the long-term course of depression, especially the time and rates of recurrence. Moreover, the natural history has rarely been compared between early- and late-onset depression, mild and severe depression. As the mechanism of PSD at the acute stage following stroke may be different from later stage and depression persistence could be predicted by initial depression severity, it is important to know the differences in the courses of PSD by these two key factors.

**Added value of this study**

In the present study, we performed a population-based cohort study to demonstrate the natural history (prevalence, incidence, duration and recurrence rates) of depression up to 18-years after stroke and assess the differences by onset-time (early-onset (3-months after stroke) vs late-onset (1-year after stroke) PSD) and initial depression severity (mild vs severe PSD). We found that about 90% of incident depression occurred within 5-years after stroke. In patients with incident PSD at any time-point, about half of them recovered at the following year and two thirds of those recovered would experience depression again within 5-years following recovery. Of those not recovered at the following year, about two thirds had a duration of more than 2 years. Early- and late-onset depression presented similar natural history, while severe depression occurred earlier, had a longer duration and quicker recurrence as compared to mild depression.

**Implications of all the available evidence**

Screening for PSD could focus on those who ever depressed after 5-years since stroke in medical resource limited areas as new-onset depression was less common after this time-point. More clinical attention should be paid to patients who had a duration of depression longer than 1-year because of the high risks of experiencing persistent depression. In the design of future observational studies on PSD, follow-up up to 5-years post onset of depression with short time-interval visits (no longer than 1 year) might be cost-effective. Patients with initial high level of depressive symptoms may be most likely to benefit from closer follow-up and longer-term care of their depressive symptoms.

## Introduction

Post-stroke depression (PSD) is common after stroke and is associated with poor functional ability and increased mortality.^1^ Defining the natural history of PSD could facilitate targeted screening and tailored intervention and prevention strategies. However, current evidence on the natural history of PSD is limited to short follow-up. Our recent meta-analysis found that most studies on the natural history of PSD had a follow-up within 1-year and only one study with follow-ups beyond 5-years after stroke,^2^ which was inadequate to have a better understanding of the long-term course of depression. It has been reported that PSD could start at any point after stroke and different risk factors may be associated with different timing of onset.^3^, ^4^, ^5^, ^6^ Correspondingly, the mechanism of PSD is multifactorial, and depression at the acute stage following stroke may be pathogenically different from depression occurring later after stroke.^1^^,^^7^ As different mechanisms of PSD act at different time-point, it is interesting to know if there are any differences in the course between early- and late-onset PSD. According to the literature, initial severity of depression plays an important role in determining the depression persistence. Harris et al. found patients with high initial depressive symptom score were more likely to develop chronic symptoms^8^ and Beekman et al. suggested depression persistence could be predicted by initial depression severity.^9^ Based on the evidence, it seems that severe depression and mild depression may follow different courses. However, to the best of our knowledge, the natural history had rarely been systematically compared between mild and severe PSD.

An earlier analysis by our group in 2012 based on data from South London Stroke Register (SLSR) estimated the natural history of depression up to 15-years after stroke.^10^ The aims of the present study are to extend our earlier analysis^10^ to assess the prevalence, incidence, duration and recurrence rates of depression up to 18-years with a greater number of participants to be able to conduct key subgroup analysis with larger sample size. We specifically aim to compare: 1) early-onset (3-months after stroke) and late-onset (after 3-months after stroke) PSD; and 2) initial mild and severe PSD. Our study could facilitate targeted screening and tailored intervention and prevention strategies. As long-term follow-up is time-consuming and costly, the present study may also provide information to future design of observational studies on PSD (e.g., optimal follow-up period and frequency) in Europe.

## Methods

The SLSR is a prospective, population-based study, running continuously since 1995, which aims to recruit all patients with a first stroke in a defined geographical area of south London, within Lambeth and North Southwark boroughs.^11^ At the time of the 2011 UK census, the source population of the SLSR was 357 308 (50.4% men), comprising 56% White, 25% Black (14% Black African, 7% Black Caribbean, and 4% Other Black), and 18% Other ethnic groups.^12^ Stroke was defined using the World Health Organization ICD-10 criteria,^13^ with subtypes (ischemic and haemorrhagic stroke) defined by a study stroke physician, with reference to symptoms, neuroimaging and other test results. Data from patients, registered in the SLSR between 1-1-1995 and 31-07-2019, and followed up until 31-10-2019 were used.

### Standard protocols, registrations, and patient consent

All patients or their relatives gave written, informed consent to their participation in the study. SLSR was conducted in accordance with the Declaration of Helsinki, and ethical approval was granted by the ethics committees of Guy’s and St Thomas’ NHS Foundation Trust, King’s College Hospital Foundation Trust, Queens Square, and Westminster Hospital (London).

### Participants and recruitment

Participants were registered during the acute phase of their first stroke, and when possible, the initial assessment was conducted within 48 h of referral to the SLSR. The baseline data included socio-demographic factors, socioeconomic status, stroke subtype (ischemic or haemorrhagic stroke), living condition (living alone or living with someone), physical disability, stroke severity, self-reported pre-stroke depression, and cognitive function. Demographic data included age, sex (male or female) and ethnicity (white, black, and other ethnicity). Index of multiple deprivation (IMD) was used to rank the socioeconomic status with higher score indicating more deprived. Physical disability was assessed using the Barthel index (BI)^14^: scores of 0–14 was categorised as severe disability. Stroke severity was measured by the National Institutes of Health Stroke Scale (NIHSS): scores >20 were categorised as severe stroke.^15^ Cognitive function was assessed with the Mini-Mental State examination (MMSE)^16^ prior to 2000 and with the Abbreviated Memory Test (AMT)^17^ after 2000. MMSE scores <24 or AMT<8 were considered cognitive impairment.^16^^,^^17^

### Follow-up

Follow-up data were collected by validated postal or face-to-face instruments with patients and/or their careers, depending on the capacity of patient. All interviewers went through regular standardized training in the use of the various scales. After initial assessment, participants underwent follow-up interviews at 3-months, 1-year, then annually after stroke until Mar 2014. Since April 2014 participants have been followed up at 3-months, 1 year, 5 years, 15-years then annually. Patients who lost to follow-up at one time-point would be contacted again for the following assessment. Depression was assessed using the Hospital Anxiety and Depression Scale (HADS) at follow-up.^18^ HADS is well validated and widely used in stroke patients and showed a good performance both in a face-to-face interview and self-administration (Cronbach α > 0.80; optimum performance when HAD subscale scores >7 is used to identify depression; sensitivity, 0.73; specificity, 0.82).^19^ Despite its good performance, HADS is not a diagnostic scale but a screening tool that indicates risk of depression. However, we use the term depression in patients with scores >7 in this paper for succinctness. HADS is also proved to be valid measures of severity of the emotional disorder (correlations of the depression subscale scores and the psychiatric ratings r = 0.70).^18^ Higher cut-off values (cut-off value of 11 or higher) could be used to identify people with higher depression symptom.^20^ Therefore, scores at 8–10 indicate lower level of depression symptoms and scores ≥ 11 indicate higher level of depression symptoms. However, we use the term ‘mild depression’ in patients with scores at 8–10 and term ‘severe depression’ in patients with scores ≥ 11 for succinctness. HADS scores were routinely collected from 1997. Patients registered in 1995 and 1996 did not have their first HAD scale assessment until 1997. The follow-up interview included many other questionnaires in addition to the HADS. Usually, before the fieldworkers reached the HADS, they were able to judge the degree to which the patient may have cognitive impairment or aphasia. If it was evident that the patient was very confused, had difficulties in understanding the questions, or was unable to clearly express their thoughts, their responses would be invalid. Also, if the patient’s speech was so poor that they were unable to effectively answer the HADS or patients were becoming distressed by the pressure of trying to speak when they were struggling to get the words out, the questions would not be asked, and the test would be terminated. Since depression cannot reliably be assessed by proxy, those with severe cognitive or communication problems that the fieldworker judged would give invalid responses were not assessed. Self-reported antidepressants (tricyclic antidepressants, selective serotonin reuptake inhibitors (SSRIs) or other antidepressants) were also recorded at each follow-up. For patients with possible depression during the interview, fieldworkers would raise the issue of low mood and ask them if they have seen a GP or have sought counselling during the interview. If the patients haven’t, the fieldworkers would encourage the participant to contact their GP.

### Statistical analyses

The prevalence of PSD was calculated among survivors assessed at each time-point. The cumulative incidence of depression was the proportion of patients with depression at one or more time-points among patients assessed for depression at any time-points. The proportion of patients depressed at 3-months or 1-year who recovered each year was calculated among patients with complete follow-up until each time-point. Finally, to estimate the depression recurrence, the proportion of patients depressed at 3-months, recovered at 1-year and experienced depression again in the following assessment was calculated among patients assessed for depression (recurrence) after recovery at any time-points. Chi-squared test (categorical variables) and t-test (continuous variables) were used to compare baseline characteristics of survivors completing and not completing HADS. Comparisons of recovery and recurrence rates between mild and severe depression were performed using two proportional Z test. Logistic regression models adjusted for age, sex, ethnicity, and stroke severity were used to explore the associations between depression severity and recovery.

Firstly, all estimates were obtained from patients with complete data (complete case [CC] analysis). However, estimates obtained from CC analysis may produce bias if the characteristics of excluded patients were statistically different from the included. Therefore, secondly, a weighted estimation using inverse probability weighting (IPW) was performed with the aim to alleviate the “selection bias”, giving each individual’s data a weight inversely proportional to their probability of selection.^21^ To weight the probability of being complete, a binary indicator of completeness (0 = missing, 1 = complete) was created for each estimate (prevalence, incidence, recovery and recurrent rates) at each time-point. The missing refers to all the patients who didn’t complete the HADS, including patients lost to follow-up and death. A logistic regression model was conducted to identify possibility of completeness. Variables included in the models were those considered to be associated with the development of PSD: age, sex, ethnicity, socioeconomic status, stroke subtype, living condition, physical disability, stroke severity, self-reported pre-stroke depression and cognitive function. The inverse of the predicted probability of being a CC was calculated and applied to individuals with available data. Finally, estimates were calculated on weighted data. As IPW may introduce error with high weights, weight truncation was used to deal with large weights.^21^ Based on the histogram of the weights generated from each logistic regression model, a maximum weight (97.5% upper limit) is chosen and all weights greater than this were set equal to it. All statistical analyses were conducted with the use of R software version 4.1.2 (Free Software Foundation).

To test the robustness of our results, three sensitivity analyses were conducted. First, there is no specific definition of late-onset depression. In addition to analyses of depression at 1-year after stroke, we further estimated recovery and recurrence rates in patients with incident PSD at 2-years or 3-years after stroke. Second, as the follow-up frequency changed since Mar 2014, patients recruited between 2011 and 2019 didn’t have depression assessed yearly. We conducted one sensitivity analysis which only included patients registered before Mar 2010 so that all the participants were offered yearly follow -up to 5-years after stroke. Third, history of depression before stroke is an established risk factor for PSD. All the estimates were repeated in patients without pre-stroke depression. Fourth, treatment for depression may have an effect on the duration of PSD. We conducted a further sensitivity analysis that excluded patients using antidepressants at any time-points.

### Role of funding source

This project is funded by the National Institute for Health and Care Research Programme (NIHR) under its Programme Grants for Applied Research (NIHR202339) and is supported by the NIHR Applied Research Collaboration (ARC) South London at King’s College London NHS Foundation. Author LL received financial support from China Scholarship Council PhD Scholarship (CSC No. 202108310074). The study funders had no role in study design, data collection, data analysis, data interpretation, or the writing of the manuscript.

## Results

### Sample description

Between 1st January, 1995 and 31st July, 2019, the SLSR recruited 6641 stroke survivors and 3864 patients were assessed for depression at one or more time-points during the 18-years follow-up. The proportion for male was 55.4% and the median age was 68.0 (20.4) years. White ethnicity comprised 62.5% and Black ethnicity comprised 29.7%. The number of patients assessed for depression at each time-point ranged from 2293 at 1-year to 145 at 18-years after stroke. The number of patients registered, completing HADS, died and lost to follow-up at each time-point are presented in Supplementary Fig. S1. Excluded patients were older and more likely to be cognitively impaired at several time-points. The proportion of severe stroke in included patients ranged from 1.0% to 3.3% across time-points, which was lower than the excluded patients (ranged from 10.8% to 17.0%). Excluded patients were more deprived (had higher IMD scores) than those included at two time-points (mean IMD scores: 35.3 ± 9.8 vs 34.3 ± 9.9 at 3-months; 35.3 ± 9.8 vs 34.2 ± 10.0 at 1-year after stroke) (Supplementary Table S1).

### Natural history of PSD

**Fig. 1:**
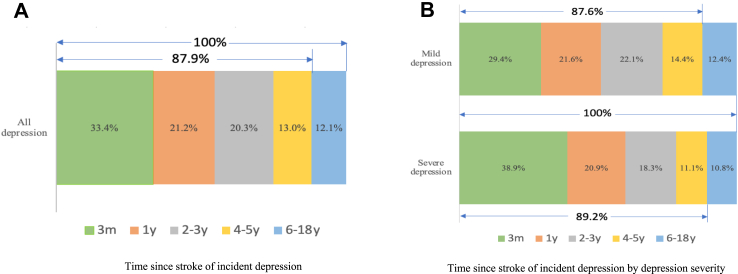
**Time since stroke of incident depression.** Notes: Part A: Among all the incident depression during the 18-years follow-up, 33.4% (95% CI: 31.5–35.4) occurred within 3-months and 87.9% (86.5–89.2) occurred within 5-years after stroke. Part B: Among all the incident mild depression during the 18-years follow-up, 29.4% (95% CI: 27.0–32.0) occurred within 3-months and 87.6% (85.7–89.3) occurred within 5-years after stroke. Among all the incident severe depression during the 18-years follow-up, 38.9% (95% CI: 35.8–42.0) occurred within 3-months and 89.2% (87.1–91.1) occurred within 5-years after stroke.

**Fig. 2:**
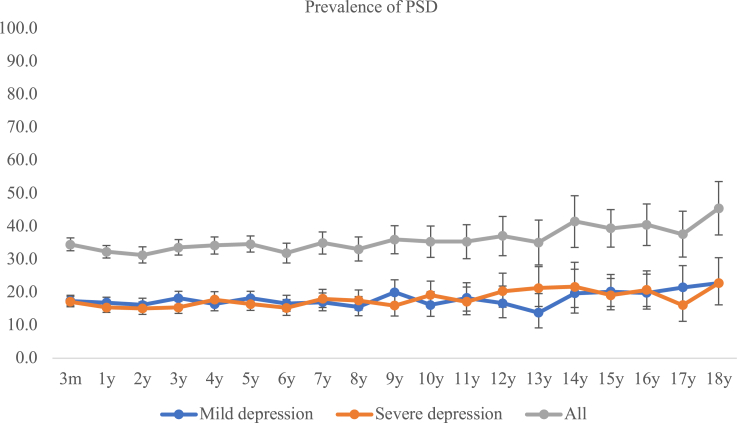
**Prevalence (%) of depression up to 18 years after stroke**.

A total of 2295 patients had depression at some time-points during the follow-up, and 1569 were not depressed at any-time points. Cumulative incidence was 59.4% (95% CI 57.8–60.9) on CC analysis and 60.4% (58.9–62.0) using IPW. Of the 2295 incident cases, 33.4% (31.5–35.4) occurred within 3-months, 54.6% (52.5–56.6) occurred within 1-year and 87.9% (86.5–89.2) occurred within 5-years after stroke (Fig. 1). The prevalence of depression ranged from 31.3% (28.9–33.8) to 45.5% (37.4–53.6) (Fig. 2 and Supplementary Table S2). Among patients with depression at 3-months, 46.6% (42.1–51.2) first recovered at 1-year, 20.3% (15.5–25.9) at 2-years and less than 5% after 5-years since occurrence (Table 1). Similar recovery rates were observed in patients with incident PSD at 1-year after stroke (Table 2). The number of patients with complete follow-up and those who died or were lost to follow-up at each time-point are presented in Supplementary Table S3. Of patients with depression at 3-months and recovered at 1-year after stroke (n = 221), 135 patients were assessed for depression (recurrence) during the follow-up and 90 patients experienced depression again. The cumulative recurrence rates during the 18-years were 66.7% (58.0–74.5), of which 94.4% (87.6–98.2) occurred within 5-years since recovery (Table 3). Of patients with depression at 3-months and not recovered at 1-year after stroke, 95 patients had complete follow-up at 2-years after stroke and 58 of them (71.6% (61.4–71.6)) were still depressed at 2-years after stroke. In patients with depression at 1-year and recovered at the following year, similar recurrence rates were obtained (Table 4).

**Table 1:** Recovery in patients with PSD at 3-months after stroke.

Recovery time (time since stroke)	Recovery time (time since depression)	Patients with depression at 3-months with complete follow-up	Patients with depression at 3-months recovered for the first time	Proportion of patients with depression at 3-months recovered for the first time (95% CI)	Patients with mild depression at 3-months with complete follow-up	Patients with mild depression at 3-months recovered for the first time	Proportion of patients with mild depression at 3-months recovered for the first time (95% CI)	Patients with severe depression at 3-months with complete follow-up	Patients with severe depression at 3-months recovered for the first time	Proportion of patients with severe depression at 3-months recovered for the first time (95% CI)
1 y	1 y	474	221	46.6 (42.1–51.2)	261	148	56.7 (50.5–62.8)^a^	213	73	34.3 (27.9–41.1)^a^
2 y	2 y	246	50	20.3 (15.5–25.9)	135	31	23.0 (16.2–31.0)	111	19	17.1 (10.6–25.4)
3 y	3 y	225	31	13.8 (9.6–19.0)	128	16	12.5 (7.3–19.5)	97	15	15.5 (8.9–24.2)
4 y	4 y	200	19	9.5 (5.8–14.4)	103	11	10.7 (5.5–18.3)	97	8	8.2 (3.6–15.6)
5 y	5 y	219	33	15.1 (10.6–20.5)	112	15	13.4 (7.7–21.1)	107	18	16.8 (10.3–25.3)
6 y	6 y	116	7	6.0 (2.5–12.0)	60	1	1.7 (0.1–8.9)	56	6	10.7 (4.0–21.9)
7 y	7 y	102	4	3.9 (1.2–9.0)	63	2	3.2 (0.4–11.0)	39	2	5.1 (0.6–17.3)
8 y	8 y	82	4	4.9 (1.5–11.1)	50	2	4.0 (0.5–13.7)	32	2	6.3 (0.8–20.8)
9 y	9 y	56	1	1.8 (0.1–8.0)	32	0	0	24	1	4.2 (0.1–21.1)
10 y	10 y	51	0	0	30	0	0	21	0	0
11 y	11 y	42	1	2.4 (0.1–10.6)	25	1	4.0 (0.1–20.4)	17	0	0
12 y	12 y	28	0	0	13	0	0	15	0	0
13 y	13 y	20	0	0	7	0	0	13	0	0
14 y	14 y	12	0	0	7	0	0	5	0	0
15 y	15 y	36	2	5.6 (0.9–16.7)	20	1	5.0 (0.1–24.9)	16	1	6.3 (0.2–30.2)
16 y	16 y	36	0	0	17	0	0	19	0	0
17 y	17 y	27	0	0	16	0	0	11	0	0
18 y	18 y	19	1	5.3 (0.2–22.3)	10	0	0	9	1	11.1 (0.3–48.2)

a Two proportional Z test was used to compare the recovery rates between mild and severe depression, a means p < 0.05.

**Table 2:** Recovery in patients with incident PSD at 1-year after stroke.

Recovery time (time since stroke)	Recovery time (time since depression)	Patients with depression at 1-year with complete follow-up	Patients with depression at 1-year recovered for the first time	Proportion of patients with depression at 1-year recovered for the first time (95% CI)	Patients with mild depression at 1-year with complete follow-up	Patients with mild depression at 1-year recovered for the first time	Proportion of patients with mild depression at 1-year recovered for the first time (95% CI)	Patients with severe depression at 1-year with complete follow-up	Patients with severe depression at 1-year recovered for the first time	Proportion of patients with severe depression at 1-year recovered for the first time (95% CI)
2 y	1 y	181	92	50.8 (43.3–58.3)	107	63	58.9 (49.0–68.3)^a^	74	29	39.2 (28.0–51.2)^a^
3 y	2 y	156	37	23.7 (17.3–31.2)	100	26	24.0 (16.0–33.6)	56	11	19.6 (10.2–32.4)
4 y	3 y	132	13	9.8 (5.3–16.3)	73	5	6.8 (2.3–15.3)	59	8	13.6 (6.0–25.0)
5 y	4 y	150	26	17.3 (11.6–24.4)	86	15	17.4 (10.1–27.1)	64	11	17.2 (8.9–28.7)
6 y	5 y	78	7	9.0 (3.7–17.6)	48	4	8.3 (2.3–20.0)	30	3	10.0 (2.1–26.5)
7 y	6 y	59	3	5.1 (1.1–14.1)	38	3	7.9 (1.7–21.4)	21	0	0
8 y	7 y	42	1	2.4 (0.1–12.6)	28	1	3.6 (0.1–18.3)	14	0	0
9 y	8 y	34	3	8.9 (1.9–23.7)	23	1	4.3 (0.1–21.9)	11	2	18.2 (2.3–51.8)
10 y	9 y	28	2	7.1 (0.9–23.5)	19	2	10.5 (1.3–33.1)	9	0	0
11 y	10 y	29	2	6.9 (0.8–22.8)	18	1	5.6 (0.1–27.3)	11	1	9.1 (0.2–41.3)
12 y	11 y	23	1	4.3 (0.1–21.9)	15	1	6.7 (0.2–31.9)	8	0	0
13 y	12 y	18	0	0	11	0	0	7	0	0
14 y	13 y	10	0	0	7	0	0	3	0	0
15 y	14 y	22	0	0	14	0	0	8	0	0
16 y	15 y	22	0	0	14	0	0	8	0	0
17 y	16 y	12	0	0	6	0	0	6	0	0
18 y	17 y	8	0	0	4	0	0	4	0	0

a Two proportional Z test was used to compare the recovery rates between mild and severe depression, a means p < 0.05.

**Table 3:** Recurrence of depression in patients with PSD at 3-months and recovered at 1-year after stroke.

Recurrent Time (Time since stroke)	Recurrent Time (Time since recovery)	Patients with depression at 3-months had assessment of recurrence at any time-point	Patients with depression at 3-months had depression recurred at any time-point	Cumulative recurrence rate of depression at each time-point	Patients with mild depression at 3-months had assessment of recurrence at any time-point	Patients with mild depression at 3-months had depression recurred at any time-point	Cumulative recurrence rate of depression in patients with mild depression	Patients with severe depression at 3-months had assessment of recurrence at any time-point	Patients with severe depression at 3-months had depression recurred at any time-point	Cumulative recurrence rate of depression in patients with severe depression
2 y	1 y	102	34	33.3 (24.3, 43.4)	68	16	23.5 (14.1, 35.4)	34	18	52.9 (35.1–70.2)^a^
3 y	2 y	119	58	48.7 (39.5, 58.1)	80	33	41.3 (30.4, 52.8)	39	25	64.1 (47.2–78.8)^a^
4 y	3 y	123	76	61.8 (52.6, 70.4)	81	45	55.6 (44.1, 66.6)	42	31	73.8 (58.0–86.1)^a^
5 y	4 y	135	83	61.5 (52.7, 69.7)	91	50	54.9 (44.2, 65.4)	44	33	75.0 (59.7–86.8)^a^
6 y	5 y	135	85	63.0 (54.2, 71.1)	91	52	57.1 (46.3, 67.5)	44	33	75.0 (59.7–86.8)^a^
7 y	6 y	135	85	63.0 (54.2, 71.1)	91	53	58.2 (47.4, 68.5)	44	33	75.0 (59.7–86.8)
8 y	7 y	135	86	63.7 (55.0, 71.8)	91	56	61.5 (50.8, 71.6)	44	33	75.0 (59.7–86.8)
9 y	8 y	135	89	65.9 (57.3, 73.9)	91	57	62.6 (51.9, 72.6)	44	33	75.0 (59.7–86.8)
10 y	9 y	135	90	66.7 (58.0, 74.5)	91	57	62.6 (51.9, 72.6)	44	33	75.0 (59.7–86.8)
11 y	10 y	135	90	66.7 (58.0, 74.5)	91	57	62.6 (51.9, 72.6)	44	33	75.0 (59.7–86.8)
12 y	11 y	135	90	66.7 (58.0, 74.5)	91	57	62.6 (51.9, 72.6)	44	33	75.0 (59.7–86.8)
13 y	12 y	135	90	66.7 (58.0, 74.5)	91	57	62.6 (51.9, 72.6)	44	33	75.0 (59.7–86.8)
14 y	13 y	135	90	66.7 (58.0, 74.5)	91	57	62.6 (51.9, 72.6)	44	33	75.0 (59.7–86.8)
15 y	14 y	135	90	66.7 (58.0, 74.5)	91	57	62.6 (51.9, 72.6)	44	33	75.0 (59.7–86.8)
16 y	15 y	135	90	66.7 (58.0, 74.5)	91	57	62.6 (51.9, 72.6)	44	33	75.0 (59.7–86.8)
17 y	16 y	135	90	66.7 (58.0, 74.5)	91	57	62.6 (51.9, 72.6)	44	33	75.0 (59.7–86.8)
18 y	17 y	135	90	66.7 (58.0, 74.5)	91	57	62.6 (51.9, 72.6)	44	33	75.0 (59.7–86.8)

Notes: Among all the recurrences of depression, 94.4% (85/90) (95% CI: 87.5%–98.2%) occurred within 5-years since recovery.

a Two proportional Z test was used to compare the recurrence rates between mild and severe depression, a means p < 0.05.

**Table 4:** Recurrence of depression in patients with incident PSD at 1-year and recovered at 2-years after stroke.

Recurrent Time (Time since stroke)	Recurrent Time (Time since recovery)	Patients with incident depression at 1-year had assessment of recurrence at any time-point	Patients with incident depression at 1-year had depression recurred at any time-point	Cumulative recurrence rate of depression at each time-point	Patients with mild incident depression at 1-year had assessment of recurrence at any time-point	Patients with mild incident depression at 1-year had depression recurred at any time-point	Cumulative recurrence rate of depression in patients with mild depression	Patients with severe incident depression at 1-year had assessment of recurrence at any time-point	Patients with severe incident depression at 1-year had depression recurred at any time-point	Cumulative recurrence rate of depression in patients with severe depression
3 y	1 y	61	22	36.1 (24.2–49.4)	44	14	31.8 (18.6–47.6)	17	8	47.1 (23.0–72.2)
4 y	2 y	68	33	48.5 (36.2–61.0)	47	21	44.7 (30.2–59.9)	21	12	57.1 (34.0–78.2)
5 y	3 y	76	45	59.2 (47.3–70.4)	52	27	51.9 (37.6–66.0)	24	18	75.0 (53.3–90.2)
6 y	4 y	76	46	60.5 (48.6–71.6)	52	28	53.8 (39.5–67.8)	24	18	75.0 (53.3–90.2)
7 y	5 y	76	46	60.5 (48.6–71.6)	52	28	53.8 (39.5–67.8)	24	18	75.0 (53.3–90.2)
8 y	6 y	76	47	61.8 (50.0–72.8)	52	28	53.8 (39.5–67.8)^a^	24	19	79.2 (57.8–92.9)^a^
9 y	7 y	77	49	63.6 (51.9–74.3)	52	30	57.7 (43.2–71.3)	24	19	79.2 (57.8–92.9)
10 y	8 y	77	50	64.9 (53.2–75.5)	53	31	58.5 (44.1–71.9)	24	19	79.2 (57.8–92.9)
11 y	9 y	77	50	64.9 (53.2–75.5)	53	31	58.5 (44.1–71.9)	24	19	79.2 (57.8–92.9)
12 y	10 y	77	50	64.9 (53.2–75.5)	53	31	58.5 (44.1–71.9)	24	19	79.2 (57.8–92.9)
13 y	11 y	77	50	64.9 (53.2–75.5)	53	31	58.5 (44.1–71.9)	24	19	79.2 (57.8–92.9)
14 y	12 y	77	50	64.9 (53.2–75.5)	53	31	58.5 (44.1–71.9)	24	19	79.2 (57.8–92.9)
15 y	13 y	77	51	66.2 (54.6–76.6)	53	32	60.4 (46.0–73.5)	24	19	79.2 (57.8–92.9)
16 y	14 y	77	51	66.2 (54.6–76.6)	53	32	60.4 (46.0–73.5)	24	19	79.2 (57.8–92.9)
17 y	15 y	77	51	66.2 (54.6–76.6)	53	32	60.4 (46.0–73.5)	24	19	79.2 (57.8–92.9)
18 y	16 y	77	51	66.2 (54.6–76.6)	53	32	60.4 (46.0–73.5)	24	19	79.2 (57.8–92.9)

Notes: Among all the recurrences of depression, 90.2% (46/51) (95% CI: 78.6–96.7) occurred within 5-years since recovery.

a Two proportional Z test was used to compare the recurrence rates between mild and severe depression, a means p < 0.05.

### Mild vs severe depression

The cumulative incidence of mild depression was 34.0% (32.5–35.6) on CC analysis and 33.0% (31.5–34.5) using IPW, while severe depression was 25.4% (24.0–26.8) on CC analysis and 26.9% (25.5–28.3) using IPW. Two proportional Z test showed the difference in cumulative rates (8.7% (6.6–10.7)) was statistically significant (p < 0.01). Among all the incident mild depression, 29.4% (27.0–32.0) occurred within 3-months and 87.6% (85.7–89.3) occurred within 5-years after stroke, whereas 38.9% (35.8–42.0) of severe depression occurred within 3-months and 89.2% (87.1–91.1) within 5-years after stroke (Fig. 1). The prevalence of mild and severe depression was similar at about 17% each at any time-point up to 18-years (Fig. 2 and Supplementary Table S4).

### Early-vs late-onset depression

Among patients with mild depression at 3-months after stroke, 56.7% (50.5–62.8) first recovered at 1-year, while in patients with severe depression, 34.3% (27.9–41.1) recovered at 1-year (Table 1). Mild depression had higher recovery rate than severe depression (22.4% (13.3–31.6), p < 0.01) (Supplementary Table S5). 58.9% (49.0–68.3) of patients with mild depression at 1-year after stroke had recovered at the following time-point, compared to 39.2% (28.0–51.2) with severe depression (difference: 19.7% (4.0, 35.4), p < 0.01) (Table 2 and Supplementary Table S5). Severe depression at 3-months after stroke had significantly lower rate of recovery at 1-year after stroke than mild depression (OR 0.43,95% CI 0.29–0.63) (Supplementary Table S6). Of patients with depression at 3-months and recovered at 1-year after stroke (N = 221), 148 experienced mild depression and 73 experienced severe depression at 3-months. A total of 91 patients with mild and 44 patients with severe depression were assessed for depression recurrence during the study period. The cumulative recurrence rates in patients with mild depression at 3-months and recovered at 1-year after stroke ranged from 23.5% (14.1–35.4) within 1-year to 62.6% (51.9–72.6) at 18-years, while in patients with severe depression, the rates rose from (52.9% (35.1–70.2) to 75.0% (59.7–86.8). Mild depression had higher recurrence rates within 5-years since recovery, but the cumulative recurrence rates during the 18-years follow-up were similar between mild and severe depression (Table 3 and Supplementary Table S7). In patients with incident PSD at 1-year and recovered at 2-years after stroke, similar differences in the recurrence by depression severity (at 1-year) were observed (Table 4 and Supplementary Table S7).

Weighted and CC estimates of prevalence, cumulative incidence, duration and recurrence rates were consistent at all time-points (Supplementary Tables S2–S4, and S8).

### Sensitivity analyses

When compared to patients with early-onset depression, similar recovery and recurrence rates were observed in patients with incident PSD at 2-years or 3-years after stroke (Supplementary Tables S3 and S8). 4608 patients were registered between 1995 and 2010 and 2601 patients were assessed for depression at some-point (Supplementary Fig. S2). The cumulative incidence of PSD was 62.4% (60.5–64.3) on CC analysis (64.8% (62.9–66.6) on IPW) (Supplementary Fig. S3). The prevalence (Supplementary Table S9), recovery (Supplementary Table S10) and recurrence rates (Supplementary Table S11) in this sensitivity analysis were similar to those in the main analyses. A total of 3581 patients were assessed for PSD at some time-points after excluding patients with pre-stroke depression (n = 562). The cumulative incidence of PSD in patients without pre-stroke depression was 58.4% (56.8–60.0) on CC analysis (58.8% (57.1–60.4) on IPW). Results for prevalence, recovery and recurrence rates in patients without pre-stroke depression were similar to the main analysis (Supplementary Fig. S4 and Tables S12–S14). A total of 858 patients were prescribed antidepressants at any time-points. Among the 5783 patients who did not take anti-depressants during the follow-up, 3081 patients were assessed for depression and 1644 were depressed at more than one time-point. The cumulative incidence of PSD in patients without antidepressants was 53.4% (51.6–55.1) on CC analysis (55.5% (54.2–56.9) on IPW), which was lower than that in the full sample (p < 0.01) (Supplementary Fig. S5). The prevalence of PSD in patients without antidepressants ranged from 26.3% (22.4–30.6) to 37.3% (25.2–39.4), which was also lower than that in the full sample (Supplementary Table S15). In patients took antidepressants, the prevalence ranged from 45.8% (34.0–58.0) to 69.2% (52.4–83.0) across time-points (Supplementary Table S16). The recovery rate at 1-year after stroke was 53.2% (47.5–58.8) (Supplementary Table S17). The recurrence rates in these patients are similar to those in the full sample (Supplementary Table S18).

## Discussion

The analyses showed that PSD affected more than half of stroke survivors at some point, with about 90% of depressed patients having their first episode of PSD within 5-year post stroke. Prevalence of PSD ranged from 31% to 45%, with half experiencing severe depression and half experiencing mild depression. Early- and late-onset showed similar natural history: recovery decreased over time, with the highest recovery rate observed within 1-year since depression and those who were not recovered had a high risk of being persistent depression. About two thirds of patients recovered from PSD would experience recurrence during the follow-up, of which over 90% occurred within 5-years since recovery. Severe depression tended to occur earlier, had a longer duration and quicker recurrence than mild depression.

Previous studies reported the cumulative incidence of PSD within 1-year after stroke ranged from 25% to 55%^22^, ^23^, ^24^ and about two thirds of the incident cases occurred within 3-months. However, depression occurring beyond 1-year after stroke is not rare and has been rarely investigated.^2^ In the present study, we found 60% of stroke survivors would experience depression at some point during the 18-years follow-up, suggesting the overall incidence of PSD may be underestimated from studies with shorter follow-up. Moreover, the present long-term follow-up study found very few incident cases occurred after 5-years post stroke. Our earlier study reported that the cumulative incidence during 5-years and 15-years post-stroke was 52% and 55% respectively,^9^^,^^25^ which didn’t differ greatly to that during 18-years, confirming our observation that most of the new-onset depression occurred within 5-years after stroke. This is an important finding as it may help to better allocate the healthcare in medical resources limited area: routine screen for depression should be provided to all stroke survivors within 5-years after stroke, whereas more medical resource could be allocated to those who ever depressed in the long term in monitoring for recurrence.

Half of the patients with PSD at 3-months recovered from depression at 1-year, which is similar to previous studies.^9^^,^^22^^,^^23^^,^^26^ However, the recovery in patients with late-onset depression has rarely been reported. The present findings complement and extend previous studies on the recovery of PSD by showing that late-and early-onset PSD showed similar course: most remission occurred within 1-year of onset and recovery was rare after 5-years. Those who had not recovered at 1-year had high risks of becoming persistently depressed. Routine screen for PSD is recommended by national guidelines, but there is no agreement on the optimal time to screen for PSD.^27^, ^28^, ^29^, ^30^ We recommended that screening for PSD could start within the first 3–6 months after stroke, with short time-intervals between two assessments (no longer than 1 year) and more attention to those had a duration longer than 1 year. It is well known that PSD has high recurrence, but the exact rates and timing of recurrence has been scarcely investigated. The present study showed that almost two thirds of patients who recovered from incident depression would experience recurrence and almost all recurrence occurred within 5-years after initial recovery. Our results overall suggested longer-term depressive status may be established by 5-years post onset as further recovery or recurrence was rare beyond this point. Therefore, In the design of future observational studies on PSD, follow-up up to 5-years post onset of depression, and with short time-intervals (no longer than 1 year) may be cost-effective.

The SLSR does not have a control arm, therefore it was not possible to know whether estimates of depression were different from the ones after any acute illness or the ones in general population. One previous study^31^ observed that the prevalence of PSD (measured at 6-months after stroke) were significantly higher in stroke survivors than control subjects matched for age and sex (37.9% vs 9.5%). Mayman et al. reported that patients with stroke were ≈50% more likely than patients with myocardial infarction to develop depression during the 1.5-year follow-up period.^32^ Compared to our estimate of cumulative incidence of PSD in stroke patients (∼60%), studies observing general population reported a lower incidence of depression between 13% and 17% during patient’s lifetime.^33^^,^^34^ The overall cumulative recurrence estimate was 42% at 20 years in the general population,^35^ which is lower than our estimate of 2/3 in stroke survivors. However, it’s hard to say if or to what extend depression is related to stroke after such a long time. Future long-term follow up studies with control arms were needed to address this question.

Thirty percent of all incident mild depression occurred within 3-months after stroke. This was 10% lower than severe depression, suggesting severe depression tended to have earlier onset than mild depression. The stable prevalence of one third was generally in line with previous literature,^36^^,^^37^ but the prevalence of mild vs severe depression demonstrated inconsistent results. Burvill et al.^38^ found major depression presented a prevalence of 15% (11–19%), while minor of 8% (5%–11%) at 4-months after stroke in 294 patients from the Perth Community Stroke Study. The differences can be attributed partly to heterogeneity in the assessment methods, differences in follow-up time, as well as different sample size. The recovery rates in patients with mild depression was significantly higher than those with severe depression, especially within 1-year after occurrence. It indicates patients with severe depression had a higher risk of having persistent depression, which is consistent to previous studies that severe depression is a predictor for persistent depression.^6^^,^^7^ Mild and severe depression experienced similar recurrence rates, but severe depression experienced recurrence faster than mild one. Therefore, these patients may be more likely to benefit from closer follow-up and longer-term care of their depressive symptoms.

The estimates of incidence, prevalence and recovery rates in patients without antidepressants were lower than those in the full sample analysis. This may be due to the exclusion of some patients with depression since patients took antidepressants had much higher prevalence of PSD when compared to those didn’t take antidepressants. Further studies are warranted to evaluate the effect of antidepressants on the recovery pattern of PSD.

### Strength and limitations

The main strengths of the study are its large sample size, population-based design and long follow-up. The SLSR is a population-based register in an area which is highly diverse in terms of age, socioeconomic status, and ethnicity. A capture-recapture analysis carried out with data from SLSR concluded that 88% of the strokes occurring in the study area were being registered.^39^ The long follow-up enables us to evaluate the course of PSD over 5-years after stroke, providing recommendations to future study design in the follow-up frequency and follow-up period. Secondly, different mechanisms of PSD act at different time-points after stroke.^1^^,^^6^ In our study, depression was assessed at multiple time-points, providing longitudinal data to assess time-dependent differences in the natural history of PSD. Moreover, we evaluated the variations in the natural history based on the onset-time by comparing the recovery and recurrence rates of patients with depression at 3-months to those at 1-year after stroke and confirmed our findings with all the estimates preformed in patients with depression at 2-years and 3-years after stroke. All these analyses made our observations more reliable.

There are several limitations in the present study. First, as in almost all cohort studies, there are missing data in the follow-ups. This was partially because of the difficulty in following up patients for such long time as well as the high mortality in stroke patients. In the present study, IPW was used to account for missing data and the estimates from IPW analysis were consistent with those with complete analysis. However, selection bias may still be present as IPW models may not fully account for this. Second, patients with severe impairment were more likely to be excluded in the analysis due to their inability to participate in the depression assessment. For example, the proportion of severe stroke in excluded patients (∼12%) was higher than included patients (∼2%). Therefore, the present study was more likely to include patients with mild and moderate troke, which may preclude generalization of our findings to all the stroke survivors. Third, the number included in the analysis beyond 10-years after stroke was small due to the high mortality in stroke patients (also aging patients) and the difficulties in following-up for patients for such long time. The results from CC analysis in later years may be less robust and IPW may also introduce bias due to the small number of complete cases to build a stable model. Therefore, the estimates beyond 10-years after stroke should be read with caution. Fourth, depression is not diagnosed with clinical interview. Although HADS is a recognized screening tool for PSD, the gold standard for diagnosis remains through clinician-administered structured clinical interviews.^40^ Fifth, even though the fieldworkers used the same criteria to assess patients’ abilities to give valid answer to HADS, we cannot exclude the possibility of some variations in different fieldworkers’ application process during the long follow-ups. This could potentially lead to some selection bias in either direction: 1) Excluded patients with mild stroke/cognitive impairment who could give valid answer to HADS 2) Included patients with severe stroke/aphasia who provided invalid answers. These may result in overestimate or underestimate depression. Even though patients with severe stroke comprised a very small proportion of patients included in the analysis, we could not rule out the possible bias. Another limitation is that the follow-up frequency changed since 2014, which resulted in the higher proportion of missing data in several time-points. However, we performed a sensitivity analysis in patients registered before 2010, all of whom were offered yearly follow -up to 5-years after stroke, and the results were similar to the main analysis. Finally, we used HADS at all the time-points after stroke. However, the optimal screening tool may vary by time since stroke. Currently, the ideal scale and whether different scales are needed at different time points poststroke is unknown. Further research is needed to define the optimal screening tool in consideration of time since stroke.

### Clinical implication

Screening for PSD could focus on those who ever depressed after 5-years since stroke in medical resource limited area. More clinical attention should be paid to patients had a duration longer than 1-year because of the high risks of experiencing persistent depression. Early-onset and late-onset showed similar natural history: both recovery and recurrence mainly occurred within 5-years since depression. In the design of future observational studies on PSD, we recommend follow-ups focus on the first 5-years after depression with short time-intervals between two assessments. Patients with a high level of depression on initial assessment tended to have longer duration and faster recurrence than those with milder symptoms. As such, these patients may be more likely to benefit from closer follow-up and longer-term care of their depressive symptoms.

### Conclusion

Prevalence of depression was stable up to 18-years after stroke, with mild and severe depression having similar frequency. Long-term depressive status may be established by 5-years post onset. Early- and late-onset depression presented similar natural history, while severe depression tended to occur earlier, had a longer duration and quicker recurrence than mild depression.

## Contributors

Lu Liu, Funding acquisition, Conceptualization, Formal analysis, Writing—original draft; Iain J Marshall, Funding acquisition, Writing—review & editing; Ruonan Pei- Formal analysis; Ajay Bhalla, Funding acquisition, Writing—review & editing; Charles DA Wolfe, Funding acquisition, Writing—review & editing; Matthew DL O’Connell, Funding acquisition, Conceptualization, Supervision, Writing—review & editing and Yanzhong Wang, Funding acquisition, Conceptualization, Supervision, Writing—review & editing.

## Data sharing statement

Because of the sensitive nature of the data collected for this study, requests to access the data set for academic use should be made to the SLSR team: https://www.kcl.ac.uk/lsm/research/divisions/hscr/research/groups/stroke/index.aspx.

## Declaration of interests

The authors declare no conflict of interest.

## References

[1] Towfighi A., Ovbiagele B., El Husseini N. (2017). American heart association stroke Council; Council on cardiovascular and stroke nursing; and Council on quality of care and outcomes research. Poststroke depression: a scientific statement for healthcare professionals from the American heart association/American stroke association. Stroke.

[2] Liu L., Xu M., Marshall I.J., Wolfe C.D.A., Wang Y., O’Connell M.D.L. (2023). Prevalence and natural history of depression after stroke: a systematic review and meta-analysis of observational studies. PLoS Med.

[3] Whyte E.M., Mulsant B.H. (2002). Post stroke depression: epidemiology, pathophysiology, and biological treatment. Biol Psychiatr.

[4] Åström M., Adolfsson R., Asplund K. (1993). Major depression in stroke patients. A 3-year longitudinal study. Stroke.

[5] Wang Z., Zhu M., Su Z. (2017). Post-stroke depression: different characteristics based on follow-up stage and gender-a cohort perspective study from Mainland China. Neurol Res.

[6] Geerlings S.W., Beekman A.T., Deeg D.J., Van Tilburg W. (2000). Physical health and the onset and persistence of depression in older adults: an eight-wave prospective community-based study. Psychol Med.

[7] Werheid K.A. (2015). Two-phase pathogenetic model of depression after stroke. Gerontology.

[8] Harris T., Cook D.G., Victor C., DeWilde S., Beighton C. (2006). Onset and persistence of depression in older people--results from a 2-year community follow-up study. Age Ageing.

[9] Beekman A.T., Deeg D.J., Geerlings S.W., Schoevers R.A., Smit J.H., Van Tilburg W. (2001). Emergence and persistence of late life depression: a 3-year follow-up of the Longitudinal Aging Study Amsterdam. J Affect Disord.

[10] Ayerbe L., Ayis S., Crichton S., Wolfe C.D.A., Rudd A.G. (2013). The natural history of depression up to 15 years after stroke: the South London stroke register. Stroke.

[11] Marshall I.J., Wolfe C., Emmett E. (2023). Cohort profile: the South London Stroke Register - a population-based register measuring the incidence and outcomes of stroke. J Stroke Cerebrovasc Dis.

[12] ONS (2011).

[13] Aho K., Harmsen P., Hatano S., Marquardsen J., Smirnov V.E., Strasser T. (1980). Cerebrovascular disease in the community: results of a WHO collaborative study. Bull World Health Organ.

[14] Mahoney F.I., Barthel D.W. (1965). Functional evaluation: the barthel index. Md State Med J.

[15] Goldstein L.B., Bertels C., Davis J.N. (1989). Interrater reliability of the NIH stroke scale. Arch Neurol.

[16] Tombaugh T.N., McIntyre N.J. (1992). The mini-mental state examination: a comprehensive review. J Am Geriatr Soc.

[17] Jitapunkul S., Pillay I., Ebrahim S. (1991). The abbreviated mental test: its use and validity. Age Ageing.

[18] Zigmond A.S., Snaith A.R. (1983). The hospital anxiety and depression scale. Acta Psychiatr Scand.

[19] Aben I., Verhey F., Lousberg R., Lodder J., Honig A. (2002). Validity of the Beck depression inventory, Hospital anxiety and Depression scale, SCL-90 and Hamilton depression rating scale as screening instruments for depression in stroke patients. Psychosomatics.

[20] Wu Y., Levis B., Sun Y., DEPRESsion screening data (DEPRESSD) HADS Group (2021). Accuracy of the Hospital Anxiety and Depression Scale Depression subscale (HADS-D) to screen for major depression: systematic review and individual participant data meta-analysis. BMJ.

[21] Seaman S.R., White I.R. (2011). Review of inverse probability weighting for dealing with missing data. Stat Methods Med Res.

[22] El Husseini N., Goldstein L.B., Peterson E.D. (2017). Depression status is associated with functional decline over 1-year following acute stroke. J Stroke Cerebrovasc Dis.

[23] Suzuki A., Mutai H, Furukawa T, Wakabayashi A, Hanihara T. (2022). The prevalence and course of neuropsychiatric symptoms in stroke patients impact functional recovery during in-hospital rehabilitation. Top Stroke Rehabil.

[24] Fournier L.E., Beauchamp J.E.S., Zhang X. (2020). Assessment of the progression of poststroke depression in ischemic stroke patients using the patient health questionnaire-9. J Stroke Cerebrovasc Dis.

[25] Ayerbe L., Ayis S., Rudd A.G., Heuschmann P.U., Wolfe C.D. (2011). Natural history, predictors and associations of depression 5 years after stroke: the South London Stroke Register. Stroke.

[26] Stokman-Meiland D.C.M., Groeneveld I.F., Arwert H.J. (2022). The course of depressive symptoms in the first 12 months post-stroke and its association with unmet needs. Disabil Rehabil.

[27] Miller E.L., Murray L., Richards L., On behalf of the American Heart Association Council on Cardiovascular Nursing and the Stroke Council (2010). Comprehensive over- view of nursing and interdisciplinary rehabilitation care of the stroke patient: a scientific statement from the American Heart Association. Stroke.

[28] Intercollegiate Stroke Working Party (2012).

[29] Stroke Foundation of New Zealand, New Zealand Guidelines Group (2010).

[30] National Stroke Foundation (2010).

[31] Kase C.S., Wolf P.A., Kelly-Hayes M., Kannel W.B., Beiser A., D’Agostino R.B. (1998). Intellectual decline after stroke: the framingham study. Stroke.

[32] Mayman N., Stein L.K., Erdman J. (2021). Risk and predictors of depression following acute ischemic stroke in the elderly. Neurology.

[33] Kessler R.C., Berglund P., Demler O. (2003). National comorbidity survey replication. The epidemiology of major depressive disorder: results from the national comorbidity survey replication (NCS-R). J Am Med Assoc.

[34] Hasin D.S., Goodwin R.D., Stinson F.S., Grant B.F. (2005). Epidemiology of major depressive disorder: results from the national epidemiologic survey on alcoholism and related conditions. Arch Gen Psychiatry.

[35] Hardeveld F., Spijker J., De Graaf R., Nolen W.A., Beekman A.T.F. (2010). Prevalence and predictors of recurrence of major depressive disorder in the adult population. Acta Psychiatr Scand.

[36] Ayerbe L., Ayis S., Wolfe C.D.A., Rudd A.G. (2013). Natural history, predictors and outcomes of depression after stroke: systematic review and meta-analysis. Br J Psychiatry.

[37] Hackett M.L., Pickles K. (2014). Part I: frequency of depression after stroke: an updated systematic review and meta-analysis of observational studies. Int J Stroke.

[38] Burvill P.W., Johnson G.A., Jamrozik K.D. (1995). Prevalence of depression after stroke: the Perth community stroke study. Br J Psychiatry.

[39] Tilling K., Sterne J.A., Wolfe C.D. (2001). Estimation of the incidence of stroke using a capture-recapture model including covariates. Int J Epidemiol.

[40] Spalletta G., Robinson R.G. (2010). How should depression be diagnosed in patients with stroke?. Acta Psychiatr Scand.

