# Supplementary material for: Natural history of depression up to 18 years after stroke: a population-based South London Stroke Register study
Source: Lancet Reg Health Eur. 2024 Mar 25;40:100882. doi: 10.1016/j.lanepe.2024.100882 (PMC11092885; doi:10.1016/j.lanepe.2024.100882)
Supplement: Supplementary Tables S1–S18 and Figs. S1–S5 [file mmc1.pdf]

Supplementary Table 1 Comparison of baseline characteristics of the survivors assessed vs not assessed with the HADS at each time-point

Follow-up	Age (Mean ± SD)	Male gender (%)	White ethnicity (%)	Ischemic stroke (%)	Living alone (%)	Severe disability (%)	Severe stroke (%)	Pre-stroke depression (%)	Cognitive impairment (%)	Socioeconomic status (IMD score: Mean± SD)
3m	67.5±14.5 vs 70.5±15.5[a]	54.7vs49.7[a]	63.3vs69.1[a]	86.9vs78.4[a]	35.3vs35.5	39.7vs59.7[a]	2.2vs17.0[a]	9.2vs13.1[a]	21.5vs30.8[a]	34.3±9.9vs35.3±9.8[a]
1y	66.9±14.3 vs 70.8±15.5[a]	55.0vs49.5[a]	64.0vs68.8[a]	86.6vs78.4[a]	34.8vs35.8	37.6vs61.4[a]	2.9vs16.3[a]	9.9vs12.8[a]	23.0vs39.8[a]	34.2±10.0vs35.3±9.8[a]
2y	66.6±14.0 vs 70.3±15.4[a]	53.8vs50.8	65.5vs67.6	86.3vs79.9[a]	33.4vs36.0	35.5vs57.0[a]	2.9vs13.6[a]	8.3vs12.5[a]	23.0vs39.8[a]	35.0±10.3vs34.9±9.7
3y	65.9±13.4 vs 70.6±15.6[a]	56.6vs49.8	69.2vs66.5	85.1vs80.1[a]	34.6vs35.7	38.9vs56.7[a]	3.3vs13.1[a]	9.5vs12.4	25.6vs39.1[a]	34.8±11.0vs35.0±9.5
4y	65.0±13.4 vs 70.6±15.4[a]	54.3vs50.7	67.7vs67.0	83.8vs80.7	32.6vs36.1	37.6vs56.1[a]	2.6vs12.9[a]	10.4vs12.0	25.6vs38.2[a]	35.0±11.0vs34.9±9.6
5y	63.8±13.5 vs 71.1±15.3[a]	57.5vs49.7[a]	63.0vs68.3[a]	83.6vs80.6	33.5vs36.0	31.3vs58.7[a]	2.6vs13.4[a]	7.9vs12.8[a]	22.1vs40.2[a]	34.5±10.2vs35.0±9.8
6y	63.5±12.8 vs 70.5±15.4[a]	56.9vs50.5[a]	67.0vs67.2	83.2vs81.0	31.3vs36.1	36.1vs55.1[a]	2.4vs11.9[a]	10.9vs11.9	24.2vs37.2[a]	34.4±11.5vs34.0±9.6
7y	63.2±12.8 vs 70.3±15.3[a]	55.7vs50.8	65.1vs67.4	80.0vs81.5	33.3vs34.7	37.1vs54.4[a]	1.4vs11.9[a]	12.2vs11.7	25.7vs36.6[a]	34.7±11.3vs35.0±9.7
8y	62.1±12.1 vs 70.3±15.3[a]	59.4vs50.5[a]	61.7vs67.7	80.2vs81.4	32.8vs35.7	38.0vs53.9[a]	2.1vs11.5[a]	12.9vs11.7	22.4vs36.7[a]	34.5±11.7vs35.0±9.6
9y	61.5±12.3 vs 70.1±15.3[a]	58.9vs50.8[a]	63.1vs67.5	80.2vs81.4	30.7vs35.8	33.0vs54.0[a]	1.3vs11.4[a]	15.1vs11.6	20.5vs36.5[a]	34.9±11.5vs34.9±9.7
10y	60.6±11.8 vs 70.1±15.2[a]	58.7vs50.9[a]	63.8vs67.4	79.0vs81.4	32.0vs35.6	32.8vs53.5[a]	2.0vs11.0[a]	14.4vs11.7	20.1vs36.0[a]	34.3±11.9vs35.0±9.7
11y	60.5±12.3 vs 70.0±15.2[a]	60.5vs50.9[a]	64.7vs67.3	77.9vs81.5	33.2vs35.5	36.2vs53.1[a]	1.9vs11.0[a]	15.1vs11.7	22.0vs35.8[a]	34.8±12.0vs34.9±9.8
12y	59.0±11.9 vs 69.9±15.2[a]	59.4vs51.1	63.5vs67.3	74.6vs81.5[a]	30.5vs35.6	38.2vs52.8[a]	1.6vs10.9	15.9vs11.7	23.9vs35.5[a]	34.7±12.1vs35.0±9.8
13y	58.4±11.8 vs 69.8±15.2[a]	61.2vs51.1[a]	61.7vs67.3	73.7vs81.5[a]	30.5vs35.5	42.4vs52.5	10.0vs10.8	17.8vs11.6	29.2vs35.2	35.1±12.3vs34.9±9.8
14y	58.6±12.0 vs 69.7±15.2[a]	61.4vs51.2[a]	67.6vs67.1	74.0vs81.5	28.8vs35.6	39.1vs52.5[a]	1.0/vs10.8	15.7vs11.7	30.9vs35.1	34.9±12.7vs34.9±9.8
15y	55.9±12.3 vs 70.1±15.0[a]	55.0vs51.2	56.1vs67.6[a]	70.3vs81.8[a]	21.0vs36.0[a]	28.6vs53.3[a]	2.5vs11.0[a]	12.8vs11.8	14.9vs36.0[a]	35.3±11.2vs34.9±9.8
16y	54.1±12.4 vs 70.1±15.0[a]	56.9vs51.2	56.1vs67.5[a]	70.4vs81.7[a]	19.3vs35.9[a]	31.9vs53.0[a]	2.3vs11.0[a]	15.5vs11.7	15.2vs35.8[a]	35.1±11.9vs34.9±9.8
17y	53.4±12.9 vs 70.0±15.0[a]	57.0vs51.2	54.6vs67.5[a]	69.8vs81.6[a]	23.4vs35.8[a]	32.4vs52.8[a]	1.7vs10.9	15.5vs11.7	11.1vs35.7[a]	35.3±12.0vs34.9±9.8
18y	52.2±11.8 vs 69.9±15.2[a]	51.3vs55.9	55.9vs67.4[a]	68.8vs81.6[a]	23.2vs35.6	34.8vs52.6[a]	3.3vs10.8	18.8vs11.7	17.1vs35.4[a]	36.0±11.5vs34.9±9.8

Note:
a: $p<0.01$

Supplementary Table 2 Prevalence of depression up to 18 years after stroke

Time since stroke	Number of patients died at each time-point	Number of patients alive at each time-point	Number of patients assessed for depression at each time-point	Number of patients with depression at each time-point	Prevalence of depression at each time-point (95%CI)	Weighted Prevalence of depression at each time-point (95% CI) [a]
3m	1497	5144	2223	768	34.5(32.6-36.5)	36.4(34.5-38.4)
1y	1937	4550	2293	740	32.3(30.4-34.2)	35.5(33.5-37.4)
2y	2201	4022	1380	432	31.3(28.9-33.8)	32.5(30.0-35.0)
3y	2477	3538	1540	518	33.6(31.3-36.0)	34.5(32.1-36.8)
4y	2646	3146	1301	445	34.2(31.6-36.8)	34.4(31.9-37.0)
5y	2802	2796	1447	501	34.6(32.2-37.1)	35.7(33.2-38.2)
6y	2916	2496	929	296	31.9(28.9-34.9)	33.0(29.9-36.0)
7y	2974	2209	772	270	35.0(31.6-38.3)	34.5(31.2-37.9)
8y	3013	1981	640	212	33.1(29.5-36.8)	32.8(29.2-36.5)
9y	3025	1713	495	178	36.0(31.7-40.2)	34.7(30.5-38.8)
10y	3024	1494	396	140	35.4(30.6-40.1)	33.2(28.5-37.8)
11y	2965	1298	328	116	35.4(30.2-40.5)	30.8(25.8-35.8)
12y	2894	1131	251	93	37.1(31.1-43.0)	32.6(26.8-38.4)
13y	2703	956	188	66	35.1(28.3-41.9)	29.1(22.6-35.6)
14y	2477	816	152	63	41.5(33.6-49.3)	34.4(26.9-42.0)
15y	2291	582	282	111	39.4(33.7-45.1)	34.0(28.4-39.5)
16y	2124	485	232	94	40.5(34.2-46.8)	35.2(29.0-41.3)
17y	1956	395	186	70	37.6(30.7-44.6)	37.3(30.4-44.2)
18y	1761	307	145	66	45.5(37.4-53.6)	38.1(30.2-46.0)

Note:
a: Prevalence calculated using inverse probability weighting.

Supplementary Table 3 Recovery in patients with post-stroke depression

A: Recovery in patients with depression at 3-months after stroke

Recovery time (time since stroke)	Recovery time (time since depression)	Patients with depression at 3-months died at each time-point	Patients with depression at 3-months lost to follow-up at each time-point	Patients with depression at 3-months with complete follow-up	Patients with depression at 3-months recovered for the first time	Proportion of patients with depression at 3-months recovered for the first time (95% CI)	Weighted Proportion of patients with depression at 3-months recovered for the first time (95% CI) [a]
1y	1y	58	236	474	221	46.6(42.1-51.2)	50.4(45.9-54.9)
2y	2y	101	421	246	50	20.3(15.5-25.9)	21.7(16.5-26.8)
3y	3y	149	394	225	31	13.8(9.6-19.0)	14.0(9.5-18.6)
4y	4y	180	388	200	19	9.5(5.8-14.4)	11.0(6.7-15.4)
5y	5y	217	332	219	33	15.1(10.6-20.5)	21.1(15.7-26.5)
6y	6y	243	409	116	7	6.0(2.5-12.0)	7.1(2.4-11.7)
7y	7y	270	396	102	4	3.9(1.2-9.0)	5.9(1.4-10.5)
8y	8y	288	398	82	4	4.9(1.5-11.1)	10.5(3.8-17.1)
9y	9y	298	414	56	1	1.8(0.1-8.0)	2.3(0.1-7.8)
10y	10y	314	403	51	0	0	0
11y	11y	327	399	42	1	2.4(0.1-10.6)	0.6(0.5-5.1) [b]
12y	12y	337	403	28	0	0	0
13y	13y	341	407	20	0	0	0
14y	14y	347	401	12	0	0	0
15y	15y	353	379	36	2	5.6(0.9-16.7)	8.5(1.7-19.6) [b]
16y	16y	354	378	36	0	0	0
17y	17y	355	378	27	0	0	0
18y	18y	357	392	19	1	5.3(0.2-22.3)	7.6(0.3-23.4) [b]

Note: A total of 768 patients experienced depression at 3-months after stroke.
Patients were assessed for depression at 3-months, 1-year, and then yearly after stroke before Mar 2014, while after Mar 2014, depression was assessed at 3 months, 1-year, 5-years, 10-years, 15-years, then yearly after stroke. Therefore, the proportion of lost to follow-up is large (actually about 1/3 patients didn't have follow-up at 2-years, 3-years,4-years,6-years etc. In order to test the robust of our results, a sensitivity analysis which only include patients recruited before Mar 2010 were performed. The proportion of lost to follow-up was about 25% (see Supplementary Tables 9-10).
a: proportion of recovery calculated using IPW.
b: The logistic model to predict the probability of being completeness may be unstable since the number of complete cases was small. The estimate with IPW analysis should be read with caution.

A1: Recovery in patients with mild depression at 3-months since stroke

Recovery Time (time since stroke)	Recovery Time (time since depression)	Patients with mild depression at 3-months died at each time-point	Patients with mild depression at 3-months lost to follow-up at each time-point	Patients with mild depression at 3-months with complete follow-up	Patients with Mild depression at 3-months recovered for the first time	Proportion of patients with mild depression at 3-months recovered for the first time (95% CI)	Weighted proportion of patients with mild depression at 3- months recovered for the first time (95% CI) [a]
1y	1y	19	107	261	148	56.7(50.5-62.8)	59.3(53.4-65.3)
2y	2y	44	208	135	31	23.0(16.2-31.0)	25.8(18.4-33.1)
3y	3y	69	190	128	16	12.5(7.3-19.5)	13.3(7.4-19.2)
4y	4y	84	200	103	11	10.7(5.5-18.3)	11.1(5-17.1)
5y	5y	107	168	112	15	13.4(7.7-21.1)	15(8.3-21.6)
6y	6y	120	207	60	1	1.7(0.1-8.9)	2.5(0.1-8.0) [b]
7y	7y	135	189	63	2	3.2(0.4-11.0)	1.9(0.2-6.7) [b]
8y	8y	146	191	50	2	4.0(0.5-13.7)	6.4(1.4-14.8) [b]
9y	9y	153	202	32	0	0	0
10y	10y	163	194	30	0	0	0
11y	11y	173	189	25	1	4.0(0.1-20.4)	1.0(0.9-8.5) [b]
12y	12y	180	194	13	0	0	0
13y	13y	183	197	7	0	0	0
14y	14y	185	195	7	0	0	0
15y	15y	188	179	20	1	5.0(0.1-24.9)	9.9(1.0-26.3) [b]
16y	16y	189	181	17	0	0	0
17y	17y	189	182	16	0	0	0
18y	18y	189	188	10	0	0	0

Notes:
a: proportion of recovery calculated using IPW.
b: The logistic model to predict the probability of being completeness may be unstable since the number of complete cases was small. The estimate with IPW analysis should be read with caution.

A2: Recovery in patients with severe depression at 3-months since stroke

Recovery Time (time since stroke)	Recovery Time (time since depression)	Patients with severe depression died at each time-point	Patients with severe depression at 3-months lost to follow-up at each time-point	Patients with severe depression at 3-months with complete follow-up	Patients with severe depression at 3- months recovered for the first time	Proportion of patients with severe depression at 3- months recovered for the first time (95% CI)	Weighted proportion of patients with severe depression at 3- months recovered for the first time (95% CI) [a]
1y	1y	39	129	213	73	34.3(27.9-41.1)	35.1(28.7-41.5)
2y	2y	57	213	111	19	17.1(10.6-25.4)	19.3(11.9-26.6)
3y	3y	80	204	97	15	15.5(8.9-24.2)	17.8(10.2-25.4)
4y	4y	96	188	97	8	8.2(3.6-15.6)	11.0(4.8-17.2)
5y	5y	110	164	107	18	16.8(10.3-25.3)	13.5(7.0-20.0)
6y	6y	123	202	56	6	10.7(4.0-21.9)	10.9(4.2-20.3)
7y	7y	135	207	39	2	5.1(0.6-17.3)	6.7(1.1-16.5) [b]
8y	8y	142	207	32	2	6.3(0.8-20.8)	6.2(0.6-17.0) [b]
9y	9y	145	212	24	1	4.2(0.1-21.1)	5.1(0.1-17.2) [b]
10y	10y	151	209	21	0	0	0
11y	11y	154	210	17	0	0	0
12y	12y	157	209	15	0	0	0
13y	13y	158	210	13	0	0	0
14y	14y	162	214	5	0	0	0
15y	15y	165	200	16	1	6.3(0.2-30.2)	6.7(0.1-23.5) [b]
16y	16y	165	197	19	0	0	0
17y	17y	166	204	11	0	0	0
18y	18y	168	204	9	1	11.1(0.3-48.2)	17.3(1.1-47.1) [b]

Notes:
a: proportion of recovery calculated using IPW.
b: The logistic model to predict the probability of being completeness may be unstable since the number of complete cases was small. The estimate with IPW analysis should be read with caution.

B: Recovery in patients with incident depression at 1-year after stroke

Recovery time (time since stroke)	Recovery time (time since depression)	Patients with depression at 1-year died at each time-point	Patients with depression at 1-year lost to follow-up at each time-point	Patients with depression at 1-year with complete follow-up	Patients with depression at 1-year recovered for the first time	Proportion of patients with depression at 1-year recovered for the first Time (95% CI)	Weighted proportion of patients with depression at 1-year recovered for the first Time (95% CI) [a]
2y	1y	39	269	181	92	50.8(43.3-58.3)	52.4(45.1-59.6)
3y	2y	81	252	156	37	23.7(17.3-31.2)	25.0(18.2-31.8)
4y	3y	114	243	132	13	9.8(5.3-16.3)	12.1(6.6-17.7)
5y	4y	135	204	150	26	17.3(11.6-24.4)	16.0(10.1-21.9)
6y	5y	156	255	78	7	9.0(3.7-17.6)	9.4(4.0-16.9)
7y	6y	176	254	59	3	5.1(1.1-14.1)	5.1(1.0-12.1) [b]
8y	7y	188	259	42	1	2.4(0.1-12.6)	1.8(0.1-8.0) [b]
9y	8y	209	246	34	3	8.9(1.9-23.7)	8.0(1.4-19.3) [b]
10y	9y	219	242	28	2	7.1(0.9-23.5)	5.6(0.3-17.0) [b]
11y	10y	227	233	29	2	6.9(0.8-22.8)	7.0(0.7-18.9) [b]
12y	11y	230	236	23	1	4.3(0.1-21.9)	2.4(0.2-12.3) [b]
13y	12y	235	236	18	0	0	0
14y	13y	238	241	10	0	0	0
15y	14y	243	224	22	0	0	0
16y	15y	244	223	22	0	0	0
17y	16y	245	232	12	0	0	0
18y	17y	248	233	8	0	0	0

Notes:
a: proportion of recovery calculated using IPW.
b: The logistic model to predict the probability of being completeness may be unstable since the number of complete cases was small. The estimate with IPW analysis should be read with caution.

B1: Recovery in patients with incident mild depression at 1-year after stroke

Recovery time (time since stroke)	Recovery time (time since depression)	Patients with depression at 1-year died at each time-point	Patients with depression at 1-year lost to follow-up at each time-point	Patients with mild depression at 1-year with complete follow-up	Patients with mild depression at 1-year recovered for the first time	Proportion of patients with mild depression at 1-year recovered for the first Time (95% CI)	Weighted proportion of patients with mild depression at 1-year recovered for the first Time (95% CI) [a]
2y	1y	21	156	107	63	58.9(49.0-68.3)	59.1(47.7-66.5)
3y	2y	36	148	100	26	26.0(17.7-35.7)	27.6(18.8-36.3)
4y	3y	57	154	73	5	6.8(2.3-15.3)	8.7(3.4-16.2)
5y	4y	68	130	86	15	17.4(10.1-27.1)	17.8(8.1-23.5)
6y	5y	81	155	48	4	8.3(2.3-20.0)	8.8(2.5-18.4)
7y	6y	93	153	38	3	7.9(1.7-21.4)	7.6(1.5-18.0) [b]
8y	7y	97	159	28	1	3.6(0.1-18.3)	2.7(0.1-11.8) [b]
9y	8y	109	152	23	1	4.3(0.1-21.9)	2.2(0.3-12.0) [b]
10y	9y	116	149	19	2	10.5(1.3-33.1)	9.6(0.8-26.5) [b]
11y	10y	121	145	18	1	5.6(0.1-27.3)	8.6(0.4-25.5) [b]
12y	11y	123	146	15	1	6.7(0.2-31.9)	3.6(0.4-18.4) [b]
13y	12y	125	148	11	0	0	0
14y	13y	126	151	7	0	0	0
15y	14y	129	141	14	0	0	0
16y	15y	130	140	14	0	0	0
17y	16y	131	147	6	0	0	0
18y	17y	133	147	4	0	0	0

Note:
a: proportion of recovery calculated using IPW.
b: The logistic model to predict the probability of being completeness may be unstable since the number of complete cases was small. The estimate with IPW analysis should be read with caution.

B2: Recovery in patients with incident severe depression at 1-year after stroke

Recovery time (time since stroke)	Recovery time (time since depression)	Patients with depression at 1-year died at each time-point	Patients with depression at 1-year lost to follow-up at each time-point	Patients with severe depression at 1-year with complete follow-up	Patients with severe depression at 1-year recovered for the first time	Proportion of patients with severe depression at 1-year recovered for the first Time (95% CI)	Weighted proportion of patients with severe depression at 1-year recovered for the first Time (95% CI) [a]
2y	1y	18	113	74	29	39.2(28.0-51.2)	38.2(27.1-49.2)
3y	2y	45	104	56	11	19.6(10.2-32.4)	20.6(10-31.2)
4y	3y	57	89	59	8	13.6(6.0-25.0)	16.2(6.8-25.6)
5y	4y	67	74	64	11	17.2(8.9-28.7)	16.2(7.2-25.3)
6y	5y	75	105	30	3	10.0(2.1-26.5)	10.5(2.3-23.7) [b]
7y	6y	83	101	21	0	0	0
8y	7y	91	100	14	0	0	0
9y	8y	100	94	11	2	18.2(2.3-51.8)	19.5(2.6-46.7) [b]
10y	9y	103	93	9	0	0	0
11y	10y	106	88	11	1	9.1(0.2-41.3)	5.3(0.4-25.4) [b]
12y	11y	107	90	8	0	0	0
13y	12y	110	88	7	0	0	0
14y	13y	112	90	3	0	0	0
15y	14y	114	83	8	0	0	0
16y	15y	114	83	8	0	0	0
17y	16y	114	85	6	0	0	0
18y	17y	115	86	4	0	0	0

Notes:
a: proportion of recovery calculated using IPW.
b: The logistic model to predict the probability of being completeness may be unstable since the number of complete cases was small. The estimate with IPW analysis should be read with caution.

C: Recovery in patients with incident depression at 2-years after stroke

Recovery time (time since stroke)	Recovery time (time since depression)	Patients with depression at 2-years died at each time-point	Patients with depression at 2-years lost to follow-up at each time-point	Patients with depression at 2-years with complete follow-up	Patients with depression at 2-years recovered for the first time	Proportion of patients with depression at 2-years recovered for the first Time (95% CI)	Weighted proportion of patients with depression at 2-years recovered for the first Time (95% CI) [a]
3y	1y	19	70	127	56	44.1(35.3-53.2)	41.5(32.9-50.0)
4y	2y	50	64	102	22	21.6(14.0-30.8)	21.7(13.7-29.6)
5y	3y	57	67	92	12	13.0(6.9-21.7)	10.3(4.1-16.5)
6y	4y	72	80	64	5	7.8(2.6-17.3)	8.0(1.4-14.7)
7y	5y	82	82	52	5	9.6(3.2-21.0)	12.1(3.3-21)
8y	6y	93	77	46	4	8.7(2.4-20.8)	10.0(1.3-18.7) [b]
9y	7y	101	82	33	0	0	0
10y	8y	107	91	18	2	11.1(1.4-34.7)	12.3(1.6-30.8) [b]
11y	9y	111	90	15	0	0	0
12y	10y	120	84	12	0	0	0
13y	11y	124	87	5	0	0	0
14y	12y	128	84	4	0	0	0
15y	13y	130	75	11	0	0	0
16y	14y	132	76	8	0	0	0
17y	15y	132	77	7	0	0	0
18y	16y	132	78	6	0	0	0

Notes:
a: proportion of recovery calculated using IPW.
b: The logistic model to predict the probability of being completeness may be unstable since the number of complete cases was small. The estimate with IPW analysis should be read with caution.

C1: Recovery in patients with incident mild depression at 2-years after stroke

Recovery time (time since stroke)	Recovery time (time since depression)	Patients with depression at 2-years died at each time-point	Patients with depression at 2-years lost to follow-up at each time-point	Patients with mild depression at 2-years with complete follow-up	Patients with mild depression at 2-years recovered for the first time	Proportion of patients with mild depression at 2-years recovered for the first Time (95% CI)	Weighted proportion of patients with mild depression at 2-years recovered for the first time (95% CI) [a]
3y	1y	6	41	87	44	50.6(39.6-61.5)	51.4(40.9-61.9)
4y	2y	28	36	70	15	21.4(12.5-32.9)	22.1(12.4-31.9)
5y	3y	33	40	61	10	16.4(8.2-28.1)	17.9(8.3-27.5)
6y	4y	41	46	47	4	8.5(2.4-20.4)	8.7(0.6-16.7) [b]
7y	5y	47	48	39	2	5.1(0.6-17.3)	4.3(0.3-12.7) [b]
8y	6y	54	45	35	2	5.7(0.7-19.2)	7.2(1.1-17.9) [b]
9y	7y	60	48	26	0	0	0
10y	8y	65	54	15	2	13.3(1.7-40.5)	14.8(2.0-36.5) [b]
11y	9y	68	55	11	0	0	0
12y	10y	72	51	11	0	0	0
13y	11y	76	54	4	0	0	0
14y	12y	79	47	4	0	0	0
15y	13y	81	41	8	0	0	0
16y	14y	83	44	7	0	0	0
17y	15y	83	42	5	0	0	0
18y	16y	83	43	4	0	0	0

Notes:
a: proportion of recovery calculated using IPW.
b: The logistic model to predict the probability of being completeness may be unstable since the number of complete cases was small. The estimate with IPW analysis should be read with caution.

C2: Recovery in patients with incident severe depression at 2-years after stroke

Recovery time (time since stroke)	Recovery time (time since depression)	Patients with severe depression at 2-years died at each time-point	Patients with severe depression at 2-years lost to follow-up at each time-point	Patients with severe depression at 2-years with complete follow-up	Patients with severe depression at 2-years recovered for the first time	Proportion of patients with severe depression at 2-years recovered for the first Time (95% CI)	Weighted proportion of patients with severe depression at 2-years recovered for the first Time (95% CI) [a]
3y	1y	13	29	40	12	30.0(16.6-46.5)	30.1(11.6-48.5)
4y	2y	22	28	32	7	21.9(9.3-40.0)	22.0(7.6-36.3)
5y	3y	24	27	31	2	6.5(0.8-21.4)	10.1(2.2-23) [b]
6y	4y	31	34	17	1	5.9(0.1-28.7)	3.9(0.2-17.8) [b]
7y	5y	35	34	13	3	23.1(5.0-53.8)	16.8(2.3-41) [b]
8y	6y	39	32	11	2	18.2(2.1-45.1)	17.9(2-44.7) [b]
9y	7y	41	34	7	0	0	0
10y	8y	42	37	3	0	0	0
11y	9y	43	35	4	0	0	0
12y	10y	48	33	1	0	0	0
13y	11y	48	33	1	0	0	0
14y	12y	49	33	0	0	--	--
15y	13y	49	30	3	0	0	0
16y	14y	49	32	1	0	0	0
17y	15y	49	31	2	0	0	0
18y	16y	49	31	2	0	0	0

Notes:
a: proportion of recovery calculated using IPW.
b: The logistic model to predict the probability of being completeness may be unstable since the number of complete cases was small. The estimate with IPW analysis should be read with caution.

D: Recovery in patients with incident depression at 3-years after stroke

Recovery time (time since stroke)	Recovery time (time since depression)	Patients with depression at 3-years died at each time-point	Patients with depression at 3-years lost to follow-up at each time-point	Patients with depression at 3-years with complete follow-up	Patients with depression at 3-years recovered for the first time	Proportion of patients with depression at 3-years recovered for the first Time (95% CI)	Weighted proportion of patients with depression at 3-years recovered for the first Time (95% CI) [a]
4y	1y	22	100	132	60	45.5(36.8-54.3)	46.9(38.4-55.4)
5y	2y	45	97	112	33	29.5(21.2-38.8)	28.5(20.1-36.8)
6y	3y	66	87	101	18	17.8(10.9-26.7)	19.1(11.4-26.8)
7y	4y	80	83	91	7	7.7(3.1-15.2)	6.0(1.1-10.8)
8y	5y	91	91	72	4	5.6(1.5-13.6)	4.4(0.9-10.3)
9y	6y	99	93	62	1	1.6(0.1-8.7)	2.2(0.1-7.3) [b]
10y	7y	113	90	51	3	5.9(1.2-16.2)	5.7(1.1-13.7) [b]
11y	8y	123	86	45	2	4.4(0.5-15.1)	5.8(0.9-14.4) [b]
12y	9y	128	96	30	1	3.3(0.1-17.2)	2.4(0.1-10.8) [b]
13y	10y	133	93	28	1	3.6(0.1-18.3)	1.9(0.2-10.0) [b]
14y	11y	140	83	31	1	3.2(0.1-16.7)	1.0(0.6-7.5) [b]
15y	12y	150	70	34	1	2.9(0.1-15.3)	6.5(0.8-17.1) [b]
16y	13y	154	74	26	0	0	0
17y	14y	159	72	23	0	0	0
18y	15y	161	72	21	0	0	0

Notes:
a: proportion of recovery calculated using IPW.
b: The logistic model to predict the probability of being completeness may be unstable since the number of complete cases was small. The estimate with IPW analysis should be read with caution.

D1: Recovery in patients with incident mild depression at 3-years after stroke

Recovery time (time since stroke)	Recovery time (time since depression)	Patients with depression at 3-years died at each time-point	Patients with depression at 3-years lost to follow-up at each time-point	Patients with mild depression at 3-years with complete follow-up	Patients with mild depression at 3-years recovered for the first time	Proportion of patients with mild depression at 3-years recovered for the first Time (95% CI)	Weighted proportion of patients with mild depression at 3-years recovered for the first Time (95% CI)[a]
4y	1y	13	54	90	49	54.4(43.6-65.0)	56.7(46.5-67)
5y	2y	24	59	74	25	33.8(23.2-45.7)	32.3(21.6-42.9)
6y	3y	37	55	65	11	16.9(8.8-28.3)	18.3(8.9-27.7)
7y	4y	49	52	56	2	3.6(0.4-12.3)	2.5(0.1-8.1)
8y	5y	57	56	44	1	2.3(0.1-12.0)	1.2(0.1-6.6) [b]
9y	6y	63	62	32	1	3.1(0.1-16.2)	4.1(0.1-13.6) [b]
10y	7y	71	55	31	1	3.2(0.1-16.7)	4.6(0.2-14.6) [b]
11y	8y	77	51	29	1	3.4(0.1-17.8)	6(0.4-17.3) [b]
12y	9y	79	58	20	1	5.0(0.1-24.9)	3.7(0.1-16) [b]
13y	10y	83	55	19	1	5.3(0.1-26.0)	2.8(0.3-14.6) [b]
14y	11y	87	52	18	1	5.6(0.1-27.3)	1.8(0.9-12.7) [b]
15y	12y	94	44	19	1	5.3(0.1-26.0)	0
16y	13y	98	46	13	0	0	0
17y	14y	100	42	15	0	0	0
18y	15y	102	42	13	0	0	0

Note:
a: proportion of recovery calculated using IPW.
b: The logistic model to predict the probability of being completeness may be unstable since the number of complete cases was small. The estimate with IPW analysis should be read with caution.

D2: Recovery in patients with incident severe depression at 3-years after stroke

Recovery time (time since stroke)	Recovery time (time since depression)	Patients with depression at 3-months died at each time-point	Patients with depression at 3-months lost to follow-up at each time-point	Patients with severe depression at 3-year with complete follow-up	Patients with severe depression at 3-year recovered for the first time	Proportion of patients with severe depression at 3-year recovered for the first Time (95% CI)	Weighted proportion of patients with severe depression at 3-year recovered for the first Time (95% CI)[a]
4y	1y	9	46	42	11	26.2(13.9-42.0)	25.3(12.1-38.4)
5y	2y	21	38	38	8	21.1(9.6-37.3)	19.6(7.0-32.2)
6y	3y	29	32	36	7	19.4(8.2-36.0)	17.2(4.9-29.6)
7y	4y	31	31	35	5	14.3(4.8-30.3)	12.0(1.2-22.8)
8y	5y	34	35	28	3	10.7(2.3-28.2)	9.1(1.4-22.2) [b]
9y	6y	36	31	30	0	0	0
10y	7y	42	35	20	2	10.0(1.2-31.7)	7.4(0.3-22.6) [b]
11y	8y	46	35	16	1	6.3(0.2-30.2)	5.5(0.1-21.4) [b]
12y	9y	49	38	10	0	0	0
13y	10y	50	38	9	0	0	0
14y	11y	53	31	13	0	0	0
15y	12y	56	26	15	1	6.7(0.2-31.9)	16.6(2.8-38.9) [b]
16y	13y	56	28	13	0	0	0
17y	14y	59	30	8	0	0	0
18y	15y	59	30	8	0	0	0

Note:
a: proportion of recovery calculated using IPW.
b: The logistic model to predict the probability of being completeness may be unstable since the number of complete cases was small. The estimate with IPW analysis should be read with caution.

Supplementary Table 4 Prevalence of depression up to 18 years after stroke by depression severity

Time since stroke	Number of patients assessed at each time-point	Number of patients with mild depression at each time-point	Prevalence of mild depression at each time-point (95%CI)	Number of patients with severe depression at each time-point	Prevalence of severe depression (at each time-point 95%CI)
3m	2223	387	17.4(15.9-19.1)	381	17.1(15.6-18.8)
1y	2293	387	16.9(15.4-18.5)	353	15.4(13.9-16.9)
2y	1380	223	16.2(14.3-18.2)	209	15.1(13.3-17.1)
3y	1540	281	18.2(16.3-20.3)	237	15.4(13.6-17.3)
4y	1301	213	16.4(14.4-18.5)	232	17.8(15.8-20.2)
5y	1447	264	18.2(16.3-20.3)	237	16.4(14.5-18.4)
6y	929	154	16.6(14.2-19.1)	142	15.3(13.0-17.8)
7y	772	131	17.0(14.4-19.8)	139	18.0(15.4-20.9)
8y	640	100	15.6(12.9-18.7)	112	17.5(14.6-20.7)
9y	495	99	20.0(16.6-23.8)	79	16.0(12.8-19.5)
10y	396	64	16.2(12.7-20.2)	76	19.2(15.4-23.4)
11y	328	60	18.3(14.3-22.9)	56	17.1(13.2-21.6)
12y	251	42	16.7(12.3-21.9)	51	20.3(15.5-25.8)
13y	188	26	13.8(9.2-19.6)	40	21.3(15.7-27.8)
14y	152	30	19.7(13.7-27.0)	33	21.7(15.4-29.1)
15y	282	57	20.2(15.7-25.4)	54	19.1(14.7-24.2)
16y	232	46	19.8(14.9-25.5)	48	20.7(15.7-26.5)
17y	186	40	21.5(15.8-28.1)	30	16.1(11.2-22.2)
18y	145	33	22.8(16.2-30.5)	33	22.8(16.2-30.5)

Supplementary Table 5 Differences in recovery rates between mild and severe depression

A: Recovery differences in patients with mild vs severe depression at 3-months after stroke

Recovery time (time since stroke)	Recovery time (time since depression)	Patients with mild depression at 3-months with complete follow-up	Patients with mild depression at 3-months recovered for the first time	Proportion of patients with mild depression at 3-months recovered for the first time (95% CI)	Patients with severe depression at 3-months with complete follow-up	Patients with severe depression at 3-months recovered for the first time	Proportion of patients with severe depression at 3-months recovered for the first time (95% CI)	Differences in the recovery rates between mild and severe depression
1y	1y	261	148	56.7(50.5-62.8)	213	73	34.3(27.9-41.1)	22.4(13.3-31.6) [a]
2y	2y	135	31	23.0(16.2-31.0)	111	19	17.1(10.6-25.4)	5.9(-5.0-16.6)
3y	3y	128	16	12.5(7.3-19.5)	97	15	15.5(8.9-24.2)	-3.0(-13-1-7.1)
4y	4y	103	11	10.7(5.5-18.3)	97	8	8.2(3.6-15.6)	2.5(-6.7-11.5)
5y	5y	112	15	13.4(7.7-21.1)	107	18	16.8(10.3-25.3)	-3.4(-13.8-7.0)
6y	6y	60	1	1.7(0.1-8.9)	56	6	10.7(4.0-21.9)	N/A
7y	7y	63	2	3.2(0.4-11.0)	39	2	5.1(0.6-17.3)	N/A
8y	8y	50	2	4.0(0.5-13.7)	32	2	6.3(0.8-20.8)	N/A
9y	9y	32	0	0	24	1	4.2(0.1-21.1)	N/A
10y	10y	30	0	0	21	0	0	N/A
11y	11y	25	1	4.0(0.1-20.4)	17	0	0	N/A
12y	12y	13	0	0	15	0	0	N/A
13y	13y	7	0	0	13	0	0	N/A
14y	14y	7	0	0	5	0	0	N/A
15y	15y	20	1	5.0(0.1-24.9)	16	1	6.3(0.2-30.2)	N/A
16y	16y	17	0	0	19	0	0	N/A
17y	17y	16	0	0	11	0	0	N/A
18y	18y	10	0	0	9	1	11.1(0.3-48.2)	N/A

Notes:
a: Two proportional Z test was used to compare the recovery rates between mild and severe depression, a means $p<0.05$.
N/A: Differences in the recovery rates and 95% CIs were not presented with the aim to avoid misleading since the number of recoveries was very small (<=2).

B: Recovery differences in patients with mild vs severe depression at 1-year after stroke

**Recovery time (time since stroke)**	**Recovery time (time since depression)**	**Patients with mild depression at 1-year with complete follow-up**	**Patients with mild depression at 1-year recovered for the first time**	**Proportion of patients with mild depression at 1year recovered for the first time (95% CI)**	**Patients with severe depression at 1-year with complete follow-up**	**Patients with severe depression at 1-year recovered for the first time**	**Proportion of patients with severe depression at 1-year recovered for the first time (95% CI)**	**Differences in the recovery rates between mild and severe depression**
2y	1y	107	63	58.9(49.0-68.3)	74	29	39.2(28.0-51.2)	19.7(4.0, 35.4) [a]
3y	2y	100	26	24.0(16.0-33.6)	56	11	19.6(10.2-32.4)	4.4(-8.5, 21.3)
4y	3y	73	5	6.8(2.3-15.3)	59	8	13.6(6.0-25.0)	-6.8(-18.7, 5.3)
5y	4y	86	15	17.4(10.1-27.1)	64	11	17.2(8.9-28.7)	0.2(-12.2, 12.8)
6y	5y	48	4	8.3(2.3-20.0)	30	3	10.0(2.1-26.5)	-1.7(-11.6, 14.9)
7y	6y	38	3	7.9(1.7-21.4)	21	0	0	N/A
8y	7y	28	1	3.6(0.1-18.3)	14	0	0	N/A
9y	8y	23	1	4.3(0.1-21.9)	11	2	18.2(2.3-51.8)	N/A
10y	9y	19	2	10.5(1.3-33.1)	9	0	0	N/A
11y	10y	18	1	5.6(0.1-27.3)	11	1	9.1(0.2-41.3)	N/A
12y	11y	15	1	6.7(0.2-31.9)	8	0	0	N/A
13y	12y	11	0	0	7	0	0	N/A
14y	13y	7	0	0	3	0	0	N/A
15y	14y	14	0	0	8	0	0	N/A
16y	15y	14	0	0	8	0	0	N/A
17y	16y	6	0	0	6	0	0	N/A
18y	17y	4	0	0	4	0	0	N/A

Notes:
a: Two proportional Z test was used to compare the recovery rates between mild and severe depression, a means $p<0.05$.
N/A: Differences in the recovery rates and 95% CIs were not presented with the aim to avoid misleading since the number of recoveries was very small (<=2).

Supplementary Table 6 Adjusted ORs and 95% CIs for the association between depression severity and recovery

A: Adjusted ORs and 95% CIs for the association between depression severity and recovery in patients with depression at 3-months and recovered at 1-year after stroke

	aOR(95%CI)	P value
**Depression severity**		
Mild depression	Ref	
Severe depression	0.43(0.29-0.63)	<0.001
**Age**		
<65y	Ref	0.007
>=65y	1.73(1.16-2.58)	
**Sex**		
Male	Ref	0.998
Female	1.00(0.68-1.46)	
**Ethnicity**		
White	Ref	0.095
Black	1.43(0.94-2.18)	
**Physical disability**		
Mild disability	Ref	0.088
Severe disability	0.68(0.44-1.06)	
**Stroke severity**		
Mild stroke	Ref	0.135
Moderate and severe stroke	0.69(0.43-1.12)	

B: Adjusted ORs and 95% CIs for the association between depression severity and recovery in patients with depression at 1-year and recovered at 2-years after stroke

	aOR(95%CI)	P value
**Depression severity**		
Mild depression	Ref	0.018
Severe depression	0.43(0.29-0.63)	
**Age**		
<65y	Ref	0.390
>=65y	0.76(0.40-1.43)	
**Sex**		
Male	Ref	0.420
Female	1.29(0.69-2.40)	
**Ethnicity**		
White	Ref	0.663
Black	1.18(0.57-2.44)	
**Physical disability**		
Mild disability	Ref	0.498
Severe disability	0.78(0.38-1.61)	
**Stroke severity**		
Mild	Ref	0.301
Moderate and severe	1.47(0.71-3.04)	

C: Adjusted ORs and 95% CIs for the association between depression severity and recovery in patients with depression at 2-years and recovered at 3-years after stroke

	aOR(95%CI)	P value
**Depression severity**		
Mild depression	Ref	0.028
Severe depression	0.36(0.15-0.90)	
**Age**		
<65y	Ref	0.561
>=65y	1.28(0.56-2.93)	
**Sex**		
Male	Ref	0.078
Female	0.48(0.21-1.09)	
**Ethnicity**		
White	Ref	0.067
Black	2.44(0.94-6.36)	
**Physical disability**		
Mild disability	Ref	0.001
Severe disability	0.18(0.07-0.48)	
**Stroke severity**		
Mild	Ref	0.660
Moderate and severe	1.24(0.48-3.18)	

D: Adjusted ORs and 95% CIs for the association between depression severity and recovery in patients with depression at 3-years and recovered at 4-years after stroke

	aOR(95%CI)	P value
**Age**		
<65y	Ref	0.465
>=65y	0.75(0.35-1.61)	
**Sex**		
Male	Ref	0.743
Female	0.88(0.41-1.88)	
**Ethnicity**		
White	Ref	0.903
Black	1.06(0.44-2.53)	
**Physical disability**		
Mild disability	Ref	0.490
Severe disability	0.73(0.30-1.78)	
**Stroke severity**		
Mild	Ref	0.311
Moderate and severe	1.62(0.64-4.12)	
**Depression severity**		
Mild depression	Ref	
Severe depression	0.25(0.10-0.61)	0.002

Supplementary Table 7 Differences in cumulative recurrence rates between mild and severe depression

A: Recurrence differences in patients with mild vs severe PSD at 3-months and recovered at 1-year after stroke

Recurrent Time (Time since stroke)	Recurrent Time (Time since recovery)	Patients with mild incident depression at 3months had assessment of recurrence at any time-point	Patients with mild incident depression at 3months had depression recurred at any time-point	Cumulative recurrence rates of depression in patients with mild depression	Patients with severe incident depression at 3months had assessment of recurrence at any time-point	Patients with severe incident depression at 3months had depression recurred at any time-point	Cumulative recurrence rates of depression in patients with severe depression	Differences in the cumulative recurrence rates between mild and severe depression
2y	1y	68	16	23.5(14.1-35.4)	34	18	52.9(35.1-70.2)	29.4(7.6, 51.2) [a]
3y	2y	80	33	41.3(30.4-52.8)	39	25	64.1(47.2-78.8)	22.8(2.4, 43.3) [a]
4y	3y	81	45	55.6(44.1-66.6)	42	31	73.8(58.0-86.1)	18.2(1.0, 35.0) [a]
5y	4y	91	50	54.9(44.2-65.4)	44	33	75.0(59.7-86.8)	20.1(2.1, 38.1) [a]
6y	5y	91	52	57.1(46.3-67.5)	44	33	75.0(59.7-86.8)	17.9(1.5, 34.2) [a]
7y	6y	91	53	58.2(47.4-68.5)	44	33	75.0(59.7-86.8)	16.8(-1.3, 34.8)
8y	7y	91	56	61.5(50.8-71.6)	44	33	75.0(59.7-86.8)	13.5(-4.5, 31.4)
9y	8y	91	57	62.6(51.9-72.6)	44	33	75.0(59.7-86.8)	12.4(-5.5, 30.3)
10y	9y	91	57	62.6(51.9-72.6)	44	33	75.0(59.7-86.8)	12.4(-5.5, 30.3)
11y	10y	91	57	62.6(51.9-72.6)	44	33	75.0(59.7-86.8)	12.4(-5.5, 30.3)
12y	11y	91	57	62.6(51.9-72.6)	44	33	75.0(59.7-86.8)	12.4(-5.5, 30.3)
13y	12y	91	57	62.6(51.9-72.6)	44	33	75.0(59.7-86.8)	12.4(-5.5, 30.3)
14y	13y	91	57	62.6(51.9-72.6)	44	33	75.0(59.7-86.8)	12.4(-5.5, 30.3)
15y	14y	91	57	62.6(51.9-72.6)	44	33	75.0(59.7-86.8)	12.4(-5.5, 30.3)
16y	15y	91	57	62.6(51.9-72.6)	44	33	75.0(59.7-86.8)	12.4(-5.5, 30.3)
17y	16y	91	57	62.6(51.9-72.6)	44	33	75.0(59.7-86.8)	12.4(-5.5, 30.3)
18y	17y	91	57	62.6(51.9-72.6)	44	33	75.0(59.7-86.8)	12.4(-5.5, 30.3)

Note:
a: Two proportional Z test was used to compare the recurrence rates between mild and severe depression, a means $p<0.05$.

B: Recurrence differences in patients with mild vs severe incident PSD at 1-year and recovered at 2-years after stroke

Recurrent Time (Time since stroke)	Recurrent Time (Time since recovery)	Patients with mild incident depression at 1-year had assessment of recurrence at any time-point	Patients with mild incident depression at 1-year had depression recurred at any time-point	Cumulative recurrence rates of depression in patients with mild depression	Patients with severe incident depression at 1-year had assessment of recurrence at any time-point	Patients with severe incident depression at 1-year had depression recurred at any time-point	Cumulative recurrence rates of depression in patients with severe depression	Differences in the cumulative recurrence rates between mild and severe depression
3y	1y	44	14	31.8(18.6-47.6)	17	8	47.1(23.0-72.2)	15.3(-16.3,46.8)
4y	2y	47	21	44.7(30.2-59.9)	21	12	57.1(34.0-78.2)	12.4(-16.5, 41.4)
5y	3y	52	27	51.9(37.6-66.0)	24	18	75.0(53.3-90.2)	23.1(-2.0, 48.1)
6y	4y	52	28	53.8(39.5-67.8)	24	18	75.0(53.3-90.2)	21.2(-3.9, 46.2)
7y	5y	52	28	53.8(39.5-67.8)	24	18	75.0(53.3-90.2)	21.2(-3.9, 46.2)
8y	6y	52	28	53.8(39.5-67.8)	24	19	79.2(57.8-92.9)	25.3(4.2, 46.5) [a]
9y	7y	52	30	57.7(43.2-71.3)	24	19	79.2(57.8-92.9)	15.3(-2.7, 45.6)
10y	8y	53	31	58.5(44.1-71.9)	24	19	79.2(57.8-92.9)	20.7(-3.3, 44.7)
11y	9y	53	31	58.5(44.1-71.9)	24	19	79.2(57.8-92.9)	20.7(-3.3, 44.7)
12y	10y	53	31	58.5(44.1-71.9)	24	19	79.2(57.8-92.9)	20.7(-3.3, 44.7)
13y	11y	53	31	58.5(44.1-71.9)	24	19	79.2(57.8-92.9)	20.7(-3.3, 44.7)
14y	12y	53	31	58.5(44.1-71.9)	24	19	79.2(57.8-92.9)	20.7(-3.3, 44.7)
15y	13y	53	32	60.4(46.0-73.5)	24	19	79.2(57.8-92.9)	20.7(-3.3, 44.7)
16y	14y	53	32	60.4(46.0-73.5)	24	19	79.2(57.8-92.9)	20.7(-3.3, 44.7)
17y	15y	53	32	60.4(46.0-73.5)	24	19	79.2(57.8-92.9)	20.7(-3.3, 44.7)
18y	16y	53	32	60.4(46.0-73.5)	24	19	79.2(57.8-92.9)	20.7(-3.3, 44.7)

Note:
a: Two proportional Z test was used to compare the recurrence rates between mild and severe depression, a means $p<0.05$.

Supplementary Table 8 Recurrence of depression after stroke

A: Recurrent depression in patients with depression at 3-months and recovered at 1-year after stroke

Recurrent time (time since stroke)	Recurrent time (time since recovery)	Patients with incident depression at 3-months had assessment of recurrence at any time-point	Patients with incident depression at 3-months had depression recurred at any time-point	Cumulative recurrence rate of depression at each time-point	Weighted cumulative recurrence rate of depression at each time-point
2y	1y	102	34	33.3(24.3-43.4)	34.3(25.1-43.5)
3y	2y	119	58	48.7(39.5-58.1)	49.8(40.8-58.8)
4y	3y	123	76	61.8(52.6-70.4)	63.7(55.2-72.2)
5y	4y	135	83	61.5(52.7-69.7)	62.9(54.8-71.1)
6y	5y	135	85	63.0(54.2-71.1)	64.3(56.2-72.4)
7y	6y	135	85	63.0(54.2-71.1)	64.3(56.2-72.4)
8y	7y	135	86	63.7(55.0-71.8)	65.0(57.0-73.1)
9y	8y	135	89	65.9(57.3-73.9)	66.9(59.0-74.9)
10y	9y	135	90	66.7(58.0-74.5)	67.7(59.8-75.5)
11y	10y	135	90	66.7(58.0-74.5)	67.7(59.8-75.5)
12y	11y	135	90	66.7(58.0-74.5)	67.7(59.8-75.5)
13y	12y	135	90	66.7(58.0-74.5)	67.7(59.8-75.5)
14y	13y	135	90	66.7(58.0-74.5)	67.7(59.8-75.5)
15y	14y	135	90	66.7(58.0-74.5)	67.7(59.8-75.5)
16y	15y	135	90	66.7(58.0-74.5)	67.7(59.8-75.5)
17y	16y	135	90	66.7(58.0-74.5)	67.7(59.8-75.5)
18y	17y	135	90	66.7(58.0-74.5)	67.7(59.8-75.5)

A1: Recurrent depression in patients with incident PSD at 3 months and recovered at 1-year after stroke by depression severity

Recurrent time (time since stroke)	Recurrent time (time since recovery)	Patients with mild incident depression at 3-months had assessment of recurrence at any time-point	Patients with mild incident depression at 3-months had depression recurred at any time-point	Cumulative recurrence rates of depression in patients with mild depression	Weighted cumulative recurrence rates of depression in patients with mild depression	Patients with severe incident depression at 1-year had assessment of recurrence at any time-point	Patients with severe incident depression at 1-year had depression recurred at any time-point	Cumulative recurrence rates of depression in patients with severe depression	Weighted cumulative recurrence rates of depression in patients with severe depression
2y	1y	68	16	23.5(14.1-35.4)	23.0(13.0-33.0)	34	18	52.9(36.2-69.7)	57.2(40.6-73.9)
3y	2y	80	33	41.3(30.4-52.8)	41.6(30.8-52.4)	39	25	64.1(49.0-79.2)	63.9(48.8-79.0)
4y	3y	81	45	55.6(44.1-66.6)	56.8(46.0-67.6)	42	31	73.8(60.5-87.1)	71.7(58.1-85.3)
5y	4y	91	50	54.9(44.2-65.4)	56.9(46.7-67.1)	44	33	75.0(62.2-87.8)	73.1(60.0-86.2)
6y	5y	91	52	57.1(46.3-67.5)	58.9(48.8-69.0)	44	33	75.0(62.2-87.8)	73.1(60.0-86.2)
7y	6y	91	53	58.2(47.4-68.5)	58.9(48.8-69.0)	44	33	75.0(62.2-87.8)	73.1(60.0-86.2)
8y	7y	91	56	61.5(50.8-71.6)	59.8(49.7-69.9)	44	33	75.0(62.2-87.8)	73.1(60.0-86.2)
9y	8y	91	57	62.6(51.9-72.6)	62.8(52.9-72.7)	44	33	75.0(62.2-87.8)	73.1(60.0-86.2)
10y	9y	91	57	62.6(51.9-72.6)	64.0(54.2-73.9)	44	33	75.0(62.2-87.8)	73.1(60.0-86.2)
11y	10y	91	57	62.6(51.9-72.6)	64.0(54.2-73.9)	44	33	75.0(62.2-87.8)	73.1(60.0-86.2)
12y	11y	91	57	62.6(51.9-72.6)	64.0(54.2-73.9)	44	33	75.0(62.2-87.8)	73.1(60.0-86.2)
13y	12y	91	57	62.6(51.9-72.6)	64.0(54.2-73.9)	44	33	75.0(62.2-87.8)	73.1(60.0-86.2)
14y	13y	91	57	62.6(51.9-72.6)	64.0(54.2-73.9)	44	33	75.0(62.2-87.8)	73.1(60.0-86.2)
15y	14y	91	57	62.6(51.9-72.6)	64.0(54.2-73.9)	44	33	75.0(62.2-87.8)	73.1(60.0-86.2)
16y	15y	91	57	62.6(51.9-72.6)	64.0(54.2-73.9)	44	33	75.0(62.2-87.8)	73.1(60.0-86.2)
17y	16y	91	57	62.6(51.9-72.6)	64.0(54.2-73.9)	44	33	75.0(62.2-87.8)	73.1(60.0-86.2)
18y	17y	91	57	62.6(51.9-72.6)	64.0(54.2-73.9)	44	33	75.0(62.2-87.8)	73.1(60.0-86.2)

B: Recurrent depression in patients with incident PSD at 1-year and recovered at 2-years after stroke

Recurrent time (time since stroke)	Recurrent time (time since recovery)	Patients with incident depression at 1-year had assessment of recurrence at any time-point	Patients with incident depression at 1-year had depression recurred at any time-point	Cumulative recurrence rate of depression at each time-point	Weighted Cumulative recurrence rate of depression at each time-point
3y	1y	61	22	36.1(24.2-49.4)	36.7(24.6-48.8)
4y	2y	68	33	48.5(36.2-61.0)	48.9(37.0-60.7)
5y	3y	76	45	59.2(47.3-70.4)	57.9(46.8-69.0)
6y	4y	76	46	60.5(48.6-71.6)	59.2(48.1-70.2)
7y	5y	76	46	60.5(48.6-71.6)	59.2(48.1-70.2)
8y	6y	76	47	61.8(50.0-72.8)	60.6(49.6-71.5)
9y	7y	77	49	63.6(51.9-74.3)	62.5(51.6-73.3)
10y	8y	77	50	64.9(53.2-75.5)	63.8(53.0-74.5)
11y	9y	77	50	64.9(53.2-75.5)	63.8(53.0-74.5)
12y	10y	77	50	64.9(53.2-75.5)	63.8(53.0-74.5)
13y	11y	77	50	64.9(53.2-75.5)	63.8(53.0-74.5)
14y	12y	77	50	64.9(53.2-75.5)	63.8(53.0-74.5)
15y	13y	77	51	66.2(54.6-76.6)	65.2(54.6-75.8)
16y	14y	77	51	66.2(54.6-76.6)	65.2(54.6-75.8)
17y	15y	77	51	66.2(54.6-76.6)	65.2(54.6-75.8)
18y	16y	77	51	66.2(54.6-76.6)	65.2(54.6-75.8)

B1: Recurrent depression in patients with incident PSD at 1-year and recovered at 2-years after stroke by depression severity

Recurrent time (time since stroke)	Recurrent time (time since recovery)	Patients with mild incident depression at 1-year had assessment of recurrence at any time-point	Patients with mild incident depression at 1-year had depression recurred at any time-point	Cumulative recurrence rates of depression in patients with mild depression	Weighted cumulative recurrence rates of depression in patients with mild depression	Patients with severe incident depression at 1-year had assessment of recurrence at any time-point	Patients with severe incident depression at 1-year had depression recurred at any time-point	Cumulative recurrence rates of depression in patients with severe depression	Weighted cumulative recurrence rates of depression in patients with severe depression
3y	1y	44	14	31.8(18.6-47.6)	32.3(18.5-46.2)	17	8	47.1(23.0-72.2)	47.3(23.5-71.0)
4y	2y	47	21	44.7(30.2-59.9)	44.3(30.1-58.5)	21	12	57.1(34.0-78.2)	58.8(37.8-79.9)
5y	3y	52	27	51.9(37.6-66.0)	52.0(38.4-65.5)	24	18	75.0(53.3-90.2)	74.3(56.8-91.8)
6y	4y	52	28	53.8(39.5-67.8)	53.9(40.4-67.4)	24	18	75.0(53.3-90.2)	74.3(56.8-91.8)
7y	5y	52	28	53.8(39.5-67.8)	53.9(40.4-67.4)	24	18	75.0(53.3-90.2)	74.3(56.8-91.8)
8y	6y	52	28	53.8(39.5-67.8)	53.9(40.4-67.4)	24	19	79.2(57.8-92.9)	77.7(61.0-94.3)
9y	7y	52	30	57.7(43.2-71.3)	56.0(42.7-69.4)	24	19	79.2(57.8-92.9)	77.7(61.0-94.3)
10y	8y	53	31	58.5(44.1-71.9)	58.1(44.8-71.4)	24	19	79.2(57.8-92.9)	77.7(61.0-94.3)
11y	9y	53	31	58.5(44.1-71.9)	58.1(44.8-71.4)	24	19	79.2(57.8-92.9)	77.7(61.0-94.3)
12y	10y	53	31	58.5(44.1-71.9)	58.1(44.8-71.4)	24	19	79.2(57.8-92.9)	77.7(61.0-94.3)
13y	11y	53	31	58.5(44.1-71.9)	58.1(44.8-71.4)	24	19	79.2(57.8-92.9)	77.7(61.0-94.3)
14y	12y	53	31	58.5(44.1-71.9)	58.1(44.8-71.4)	24	19	79.2(57.8-92.9)	77.7(61.0-94.3)
15y	13y	53	32	60.4(46.0-73.5)	60.2(47.0-73.4)	24	19	79.2(57.8-92.9)	77.7(61.0-94.3)
16y	14y	53	32	60.4(46.0-73.5)	60.2(47.0-73.4)	24	19	79.2(57.8-92.9)	77.7(61.0-94.3)
17y	15y	53	32	60.4(46.0-73.5)	60.2(47.0-73.4)	24	19	79.2(57.8-92.9)	77.7(61.0-94.3)
18y	16y	53	32	60.4(46.0-73.5)	60.2(47.0-73.4)	24	19	79.2(57.8-92.9)	77.7(61.0-94.3)

C: Recurrent depression in patients with incident PSD at 2-years and recovered at 3-years after stroke

Recurrent time (time since stroke)	Recurrent time (time since recovery)	Patients with incident depression at 2-year had assessment of recurrence at any time-point	Patients with incident depression at 2-year had depression recurred at any time-point	Cumulative recurrence rate of depression at each time-point	Weighted cumulative recurrence rate of depression at each time-point
4y	1y	37	10	27.0(13.8-44.1)	23.5(9.9-37.2)
5y	2y	42	16	38.1(23.6-54.4)	36.0(21.5-50.5)
6y	3y	44	21	47.7(32.5-63.3)	45.9(31.2-60.7)
7y	4y	44	27	61.4(45.5-75.6)	60.6(46.1-75.0)
8y	5y	44	30	68.2(52.4-81.4)	67.2(53.3-81.1)
9y	6y	44	31	70.5(54.8-83.2)	68.7(55.0-82.4)
10y	7y	44	31	70.5(54.8-83.2)	68.7(55.0-82.4)
11y	8y	44	32	72.7(57.2-85.0)	71.2(57.8-84.6)
12y	9y	44	32	72.7(57.2-85.0)	71.2(57.8-84.6)
13y	10y	44	32	72.7(57.2-85.0)	71.2(57.8-84.6)
14y	11y	44	32	72.7(57.2-85.0)	71.2(57.8-84.6)
15y	12y	44	33	75.0(59.7-86.8)	73.8(60.8-86.8)
16y	13y	44	33	75.0(59.7-86.8)	73.8(60.8-86.8)
17y	14y	44	33	75.0(59.7-86.8)	73.8(60.8-86.8)
18y	15y	44	33	75.0(59.7-86.8)	73.8(60.8-86.8)

C1: Recurrent depression in patients with incident PSD at 2-year and recovered at 3-years after stroke by depression severity

Recurrent time (time since stroke)	Recurrent time (time since recovery)	Patients with mild incident depression at 2-year had assessment of recurrence at any time-point	Patients with mild incident depression at 2-year had depression recurred at any time-point	Cumulative recurrence rates of depression in patients with mild depression	Weighted cumulative recurrence rates of depression in patients with mild depression	Patients with severe incident depression at 2-year had assessment of recurrence at any time-point	Patients with severe incident depression at 2-year had depression recurred at any time-point	Cumulative recurrence rates of depression in patients with severe depression	Weighted cumulative recurrence rates of depression in patients with severe depression [a]
4y	1y	29	8	27.6(12.7-47.2)	24.1(8.6-39.7)	8	2	25.0(3.2-65.1)	24.2(2.8-57.5)
5y	2y	33	13	39.4(22.9-57.9)	38.2(21.6-54.8)	9	3	33.3(7.5-70.1)	30.8(0.7-61.0)
6y	3y	35	18	51.4(34.0-68.6)	50.1(33.5-66.6)	9	3	33.3(7.5-70.1)	30.3(0.3-60.3)
7y	4y	35	22	62.9(44.9-78.5)	62.5(46.5-78.6)	9	5	55.6(21.2-86.3)	54.5(21.9-87.0)
8y	5y	35	25	71.4(53.7-85.4)	70.3(55.2-85.5)	9	5	55.6(21.2-86.3)	54.5(21.9-87.0)
9y	6y	35	26	74.3(56.7-87.5)	72.9(58.2-87.6)	9	5	55.6(21.2-86.3)	54.5(21.9-87.0)
10y	7y	35	26	74.3(56.7-87.5)	72.9(58.2-87.6)	9	5	55.6(21.2-86.3)	54.5(21.9-87.0)
11y	8y	35	27	77.1(59.9-89.6)	76.1(61.9-90.2)	9	5	55.6(21.2-86.3)	54.5(21.9-87.0)
12y	9y	35	27	77.1(59.9-89.6)	76.1(61.9-90.2)	9	5	55.6(21.2-86.3)	54.5(21.9-87.0)
13y	10y	35	27	77.1(59.9-89.6)	76.1(61.9-90.2)	9	5	55.6(21.2-86.3)	54.5(21.9-87.0)
14y	11y	35	27	77.1(59.9-89.6)	76.1(61.9-90.2)	9	5	55.6(21.2-86.3)	54.5(21.9-87.0)
15y	12y	35	27	77.1(59.9-89.6)	76.1(61.9-90.2)	9	6	66.7(29.9-92.5)	66.6(35.8-97.4)
16y	13y	35	27	77.1(59.9-89.6)	76.1(61.9-90.2)	9	6	66.7(29.9-92.5)	66.6(35.8-97.4)
17y	14y	35	27	77.1(59.9-89.6)	76.1(61.9-90.2)	9	6	66.7(29.9-92.5)	66.6(35.8-97.4)
18y	15y	35	27	77.1(59.9-89.6)	76.1(61.9-90.2)	9	6	66.7(29.9-92.5)	66.6(35.8-97.4)

Note:
a: Results from IPW analysis should be read with caution since the number of completeness was too small to allow for a stable logistic regression model

D: Recurrent depression in patients with incident PSD at 3-years and recovered at 4-years after stroke

Recurrent time (time since stroke)	Recurrent time (time since recovery)	Patients with incident depression at 2-year had assessment of recurrence at any time-point	Patients with incident depression at 2-year had depression recurred at any time-point	Cumulative recurrence rate of depression at each time-point	Weighted cumulative recurrence rate of depression at each time-point
5y	1y	41	11	26.8(14.2-42.9)	28.4(14.6-42.1)
6y	2y	51	20	39.2(25.8-53.9)	39.2(25.8-52.6)
7y	3y	53	24	45.3(31.6-59.6)	45.0(31.6-58.4)
8y	4y	53	27	50.9(36.8-64.9)	50.5(37.0-63.9)
9y	5y	53	28	52.8(38.6-66.7)	52.5(39.1-66.0)
10y	6y	53	30	56.6(42.3-70.2)	56.4(43.0-69.7)
11y	7y	53	30	56.6(42.3-70.2)	56.4(43.0-69.7)
12y	8y	53	30	56.6(42.3-70.2)	56.4(43.0-69.7)
13y	9y	53	30	56.6(42.3-70.2)	56.4(43.0-69.7)
14y	10y	53	31	58.5(44.1-71.9)	58.4(45.1-71.7)
15y	11y	53	32	60.4(46.0-73.5)	60.5(47.3-73.6)
16y	12y	53	32	60.4(46.0-73.5)	60.5(47.3-73.6)
17y	13y	53	32	60.4(46.0-73.5)	60.5(47.3-73.6)
18y	14y	53	33	62.3(47.9-75.2)	62.2(49.1-75.2)

D1: Recurrent depression in patients with incident PSD at 3-year and recovered at 4-years after stroke by depression severity

Recurrent time (time since stroke)	Recurrent time (time since recovery)	Patients with mild incident depression at 3-year had assessment of recurrence at any time-point	Patients with mild incident depression at 3-year had depression recurred at any time-point	Cumulative recurrence rates of depression in patients with mild depression	Weighted cumulative recurrence rates of depression in patients with mild depression	Patients with severe incident depression at 3-year had assessment of recurrence at any time-point	Patients with severe incident depression at 3-year had depression recurred at any time-point	Cumulative recurrence rates of depression in patients with severe depression	Weighted cumulative recurrence rates of depression in patients with severe depression
5y	1y	33	8	24.2(11.1-42.3)	24.7(10.0-39.5)	8	3	37.5(8.5-75.5)	39.4(5.5-73.3)
6y	2y	40	15	37.5(22.7-54.2)	36.2(21.3-51.1)	11	5	45.5(16.7-76.6)	48.0(18.5-77.5)
7y	3y	42	18	42.9(27.7-59.0)	42.2(27.2-57.1)	11	6	54.5(23.4-83.3)	55.5(26.1-84.8)
8y	4y	42	20	47.6(32.0-63.6)	46.5(31.5-61.6)	11	7	63.6(30.8-89.1)	65.2(37.1-93.4)
9y	5y	42	20	47.6(32.0-63.6)	46.5(31.5-61.6)	11	8	72.7(39.0-94.0)	74.9(49.3-99.6)
10y	6y	42	22	52.4(36.4-68.0)	51.5(36.4-66.6)	11	8	72.7(39.0-94.0)	74.9(49.3-99.6)
11y	7y	42	22	52.4(36.4-68.0)	51.5(36.4-66.6)	11	8	72.7(39.0-94.0)	74.9(49.3-99.6)
12y	8y	42	22	52.4(36.4-68.0)	51.5(36.4-66.6)	11	8	72.7(39.0-94.0)	74.9(49.3-99.6)
13y	9y	42	22	52.4(36.4-68.0)	51.5(36.4-66.6)	11	8	72.7(39.0-94.0)	74.9(49.3-99.6)
14y	10y	42	23	54.8(38.7-70.2)	54.2(39.1-69.2)	11	8	72.7(39.0-94.0)	74.9(49.3-99.6)
15y	11y	42	24	57.1(41.0-72.3)	56.8(41.8-71.8)	11	8	72.7(39.0-94.0)	74.9(49.3-99.6)
16y	12y	42	24	57.1(41.0-72.3)	56.8(41.8-71.8)	11	8	72.7(39.0-94.0)	74.9(49.3-99.6)
17y	13y	42	24	57.1(41.0-72.3)	56.8(41.8-71.8)	11	8	72.7(39.0-94.0)	74.9(49.3-99.6)
18y	14y	42	25	59.5(43.3-74.4)	58.8(43.9-73.7)	11	8	72.7(39.0-94.0)	74.9(49.3-99.6)

Note:
a: Results from IPW analysis should be read with caution since the number of completeness was too small to allow for a stable logistic regression model

Supplementary Table 9 Prevalence of depression up to 18 years after stroke (sensitivity analysis, include patients recruited Jan 1995-Mar 2010)

Time Since Stroke	Number of patients died at each time-point	Number of patients alive at each time-point	Number of patients assessed for depression at each time-point	Number of patients with depression at each time-point	Prevalence of depression at each time-point (95%CI)	Weighted Prevalence of depression at each time-point (95% CI) [a]
3m	1497	5144	1285	413	32.1(29.6-34.8)	30.6(28.1-33.1)
1y	1937	4550	1508	442	29.3(27.0-31.7)	27.4(25.1-29.6)
2y	2201	4022	1217	378	31.1(28.5-33.7)	29.8(27.2-32.3)
3y	2477	3538	1482	500	33.7(31.3-36.2)	32.5(30.1-34.9)
4y	2646	3146	1301	445	34.2(31.6-36.8)	33.1(30.6-35.7)
5y	2802	2796	1101	360	32.7(29.9-35.6)	31.0(28.2-33.7)
6y	2916	2496	929	296	31.9(28.9-34.9)	29.7(26.7-32.6)
7y	2974	2209	772	270	35.0(31.6-38.3)	34.2(30.9-37.6)
8y	3013	1981	640	212	33.1(29.5-36.8)	31.9(28.3-35.5)
9y	3025	1713	495	178	36.0(31.7-40.2)	34.8(30.6-39.0)
10y	3024	1494	396	140	35.4(30.6-40.3)	34.9(30.2-39.6)
11y	2965	1298	328	116	35.4(30.2-40.5)	37.0(31.8-42.2)
12y	2894	1131	251	93	37.1(31.1-43.0)	36.9(30.9-42.9)
13y	2703	956	188	66	35.1(28.3-41.9)	34.7(27.9-41.5)
14y	2477	816	152	63	41.5(33.6-49.3)	42.0(34.1-49.8)
15y	2291	582	137	52	38.0(29.8-46.6)	35.5(27.5-43.5)
16y	2124	485	92	42	45.7(35.2-56.4)	42.0(31.9-52.1)
17y	1956	395	59	22	37.3(25.0-50.9)	28.8(17.3-40.4)
18y	1761	307	36	17	47.2(30.4-64.5)	37.3(21.5-53.1)

Notes:
a: Prevalence calculated using inverse probability weighting

Supplementary Table 10 Recovery after depression after stroke (sensitivity analysis, include patients recruited Jan 1995-Mar 2010)

A: Recovery in patients with PSD at 3-months after stroke

Recovery Time (time since stroke)	Recovery Time (time since depression)	Patients with mild depression at 3-months died at each time-point	Patients with mild depression at 3-months lost to follow-up at each time-point	Patients with mild depression at 3-months with complete follow-up	Patients with mild depression at 3- months recovered for the first time	Proportion of patients with depression at 3-months recovered for the first time (95% CI)	Weighted proportion of patients with depression at 3- months recovered for the first time (95% CI) [a]
1y	1y	36	101	276	136	49.3(43.2-55.3)	50.5(44.6-56.4)
2y	2y	62	134	217	46	21.2(16.0-27.2)	19.6(14.3-24.9)
3y	3y	97	104	212	26	12.3(8.2-17.5)	11.9(7.5-16.2)
4y	4y	117	96	200	19	9.5(5.8-14.4)	8.8(4.9-12.7)
5y	5y	142	109	162	12	7.4(3.9-12.6)	8.2(4.0-12.4)
6y	6y	164	134	115	7	6.1(2.5-12.1)	6.2(1.8-10.6)
7y	7y	186	125	102	4	3.9(1.1-9.7)	3.6(0.1-7.2)
8y	8y	203	128	82	4	4.9(1.3-12.0)	6.7(1.3-12.1)
9y	9y	213	145	55	1	1.8(0.1-9.7)	1.9(0.1-7.2) [b]
10y	10y	229	133	51	0	0	0
11y	11y	242	129	42	1	2.4(0.1-12.6)	0.7(0.5-5.3) [b]
12y	12y	252	133	28	0	0	0
13y	13y	256	137	20	0	0	0
14y	14y	262	139	12	0	0	0
15y	15y	268	135	10	1	10.0(0.3-44.5)	12.7(0.3-38.9) [b]
16y	16y	269	138	6	0	0	0
17y	17y	270	140	3	0	0	0
18y	18y	272	141	0	0	0	0

Notes:
a: proportion of recovery calculated using IPW.
b: The logistic model to predict the probability of being completeness may be unstable since the number of complete cases was small. The estimate with IPW analysis should be read with caution.

B: Recovery in patients with incident PSD at 1-year after stroke

Recovery time (time since stroke)	Recovery time (time since depression)	Patients with depression at 1-year died at each time-point	Patients with depression at 1-year lost to follow-up at each time-point	Patients with depression at 1-year with complete follow-up	Patients with depression at 1-year recovered for the first time	Proportion of patients with depression at 1-year recovered for the first Time (95% CI)	Weighted proportion of patients with depression at 1-year recovered for the first Time (95% CI) [a]
2y	1y	30	120	152	77	50.7(42.4-58.9)	51.5(43.5-59.4)
3y	2y	62	92	148	36	24.3(17.7-32.1)	23.3(16.5-30.1)
4y	3y	86	84	132	13	9.8(5.3-16.3)	8.4(3.7-13.2)
5y	4y	102	90	110	17	15.5(9.3-23.6)	15.6(8.9-22.4)
6y	5y	118	106	78	7	9.0(3.7-17.6)	8.0(2.0-14.1)
7y	6y	134	109	59	3	5.1(1.1-14.1)	5.6(1.2-12.9)
8y	7y	146	114	42	1	2.4(0.1-12.6)	2.8(0.1-9.8) [b]
9y	8y	166	102	34	3	8.9(1.9-23.7)	7.6(1.2-18.7) [b]
10y	9y	176	98	28	2	7.1(0.9-23.5)	4.0(0.1-14.2) [b]
11y	10y	184	89	29	2	6.9(0.8-22.8)	7.0(0.7-18.8) [b]
12y	11y	187	92	23	1	4.3(0.1-21.9)	2.2(0.3-12) [b]
13y	12y	192	92	18	0	0	0
14y	13y	195	97	10	0	0	0
15y	14y	200	80	22	0	0	0
16y	15y	201	79	22	0	0	0
17y	16y	202	88	12	0	0	0
18y	17y	205	89	8	0	0	0

Notes:
a: proportion of recovery calculated using IPW.
b: The logistic model to predict the probability of being completeness may be unstable since the number of complete cases was small. The estimate with IPW analysis should be read with caution.

Supplementary Table 11 Recurrence of depression after stroke (sensitivity analysis, include patients recruited Jan1995- Mar2010)

A: Recurrent depression in patients with depression at 3-months and recovered at 1-year after stroke

Recurrent time (time since stroke)	Recurrent time (time since recovery)	Patients with incident depression at 3-months had assessment of recurrence at any time-point	Patients with incident depression at 3-months had depression recurred at any time-point	Cumulative recurrence rate of depression at each time-point	Weighted cumulative recurrence rate of depression at each time-point
2y	1y	96	33	34.4(25.0-44.8)	34.1(24.6-43.6)
3y	2y	113	56	49.6(40.0-59.1)	49.7(40.5-58.9)
4y	3y	117	74	63.2(53.8-72.0)	62.8(54.0-71.6)
5y	4y	119	78	65.5(56.3-74.0)	64.6(56.0-73.2)
6y	5y	119	80	67.2(58.0-75.6)	66.4(57.9-74.9)
7y	6y	119	80	67.2(58.0-75.6)	66.4(57.9-74.9)
8y	7y	119	81	68.1(58.9-76.3)	67.2(58.8-75.7)
9y	8y	119	84	70.6(61.5-78.6)	70.0(61.8-78.2)
10y	9y	119	85	71.4(62.4-79.3)	70.8(62.6-79.0)
11y	10y	119	85	71.4(62.4-79.3)	70.8(62.6-79.0)
12y	11y	119	85	71.4(62.4-79.3)	70.8(62.6-79.0)
13y	12y	119	85	71.4(62.4-79.3)	70.8(62.6-79.0)
14y	13y	119	85	71.4(62.4-79.3)	70.8(62.6-79.0)
15y	14y	119	85	71.4(62.4-79.3)	70.8(62.6-79.0)
16y	15y	119	85	71.4(62.4-79.3)	70.8(62.6-79.0)
17y	16y	119	85	71.4(62.4-79.3)	70.8(62.6-79.0)
18y	17y	119	85	71.4(62.4-79.3)	70.8(62.6-79.0)

B: Recurrent depression in patients with incident PSD at 1-year and recovered at 2-years after stroke

Recurrent time (time since stroke)	Recurrent time (time since recovery)	Patients with incident depression at 1-year had assessment of recurrence at any time-point	Patients with incident depression at 1-year had depression recurred at any time-point	Cumulative recurrence rate of depression at each time-point	Weighted Cumulative recurrence rate of depression at each time-point
3y	1y	56	18	32.1(20.3-46.0)	33.1(20.8-45.5)
4y	2y	63	29	46.0(33.4-59.1)	46.0(33.7-58.3)
5y	3y	64	36	56.3(43.3-68.6)	56.0(43.9-68.2)
6y	4y	64	37	57.8(44.8-70.1)	57.6(45.5-69.7)
7y	5y	64	37	57.8(44.8-70.1)	57.6(45.5-69.7)
8y	6y	64	38	59.4(46.4-71.5)	59.2(47.2-71.3)
9y	7y	64	40	62.5(49.5-74.3)	61.7(49.8-73.5)
10y	8y	65	41	63.1(50.2-74.7)	63.1(51.4-74.8)
11y	9y	65	41	63.1(50.2-74.7)	63.1(51.4-74.8)
12y	10y	65	41	63.1(50.2-74.7)	63.1(51.4-74.8)
13y	11y	65	41	63.1(50.2-74.7)	63.1(51.4-74.8)
14y	12y	65	41	63.1(50.2-74.7)	63.1(51.4-74.8)
15y	13y	65	42	64.6(51.8-76.1)	64.0(51.4-76.6)
16y	14y	65	42	64.6(51.8-76.1)	64.0(51.4-76.6)
17y	15y	65	42	64.6(51.8-76.1)	64.0(51.4-76.6)
18y	16y	65	42	64.6(51.8-76.1)	64.0(51.4-76.6)

Supplementary Table 12 Prevalence of depression up to 18-years after stroke (sensitivity analysis, excluded patients with pre-stroke depression)

Time Since Stroke	Number of patients assessed for depression at each time-point	Number of patients with depression at each time-point	Prevalence of depression at each time-point (95%CI)	Weighted Prevalence of depression at each time-point (95% CI) [a]
3m	2075	695	33.5(31.5-35.6)	31.4(29.4-33.3)
1y	2129	658	30.9(28.9-32.9)	28.5(26.6-30.4)
2y	1313	406	30.9(28.4-33.5)	29.3(26.9-31.8)
3y	1449	476	32.9(30.4-35.3)	31.8(29.4-34.2)
4y	1225	411	33.6(30.9-36.3)	32.6(30.0-35.3)
5y	1371	457	33.3(30.8-35.9)	31.5(29.1-34.0)
6y	876	275	31.4(28.3-34.6)	29.4(26.4-32.4)
7y	726	250	34.4(31.0-38.0)	33.0(29.6-36.4)
8y	605	200	33.1(29.3-37.0)	31.4(27.7-35.1)
9y	469	167	35.6(31.3-40.1)	34.8(30.5-39.1)
10y	376	133	35.4(30.5-40.4)	34.2(29.4-39.0)
11y	308	108	35.1(29.7-40.7)	36.7(31.3-42.1)
12y	231	87	37.7(31.4-44.3)	38.0(31.7-44.2)
13y	169	58	34.3(27.2-42.0)	34.2(27.0-41.3)
14y	137	54	39.4(31.2-48.1)	39.4(31.2-47.5)
15y	269	104	38.7(32.8-44.8)	36.8(31.1-42.6)
16y	219	87	32.3(26.8-38.3)	35.4(29.1-41.8)
17y	175	66	37.7(30.5-45.3)	29.7(22.9-36.5)
18y	133	60	45.1(36.5-54.0)	42.3(33.9-50.7)

Note:
a: Prevalence calculated using inverse probability weighting

Supplementary Table 13 Recovery in patients with depression after stroke (sensitivity analysis, exclude patients with pre-stroke depression)

A: Recovery in patients with depression at 3-months after stroke

Recovery Time (time since stroke)	Recovery Time (time since depression)	Patients with mild depression at 3-months died at each time-point	Patients with mild depression at 3-months lost to follow-up at each time-point	Patients with mild depression at 3-months with complete follow-up	Patients with mild depression at 3- months recovered for the first time	Proportion of patients with depression at 3- months recovered for the first time (95% CI)	Weighted proportion of patients with depression at 3- months recovered for the first time (95% CI) [a]
1y	1y	51	214	430	206	47.9(43.1-52.7)	49.6(44.9-54.3)
2y	2y	90	375	230	44	19.1(14.3-24.8)	16.9(12.1-21.8)
3y	3y	134	350	211	30	14.2(9.8-19.7)	13.1(8.5-17.6)
4y	4y	164	342	189	18	9.5(5.7-14.6)	9.2(5.0-13.3)
5y	5y	198	290	207	33	15.9(11.2-21.7)	17.1(11.9-22.2)
6y	6y	224	362	109	6	5.5(2.0-11.6)	6.3(1.7-10.9)
7y	7y	251	348	96	4	4.2(1.1-10.3)	4.0(0.1-7.9)
8y	8y	266	351	78	4	5.1(1.4-12.6)	6.9(2.4-13.5)
9y	9y	276	366	53	1	1.9(0.1-10.1)	2.3(0.1-8.0) [b]
10y	10y	292	354	49	0	0	0
11y	11y	305	351	39	1	2.6(0.1-13.5)	0.8(0.5-5.8) [b]
12y	12y	314	355	26	0	0	0
13y	13y	318	359	18	0	0	0
14y	14y	324	360	11	0	0	0
15y	15y	330	329	36	2	5.6(0.7-18.7)	7.1(1.1-17.5) [b]
16y	16y	331	329	35	0	0	0
17y	17y	332	337	26	0	0	0
18y	18y	334	343	18	1	5.6(0.1-27.3)	8.5(0.4-25.2) [b]

Notes:
a: proportion of recovery calculated using IPW.
b: The logistic model to predict the probability of being completeness may be unstable since the number of complete cases was small. The estimate with IPW analysis should be read with caution.

B: Recovery in patients with incident PSD at 1-year after stroke

Recovery time (time since stroke)	Recovery time (time since depression)	Patients with depression at 1-year died at each time-point	Patients with depression at 1-year lost to follow-up at each time-point	Patients with depression at 1-year with complete follow-up	Patients with depression at 1-year recovered for the first time	Proportion of patients with depression at 1-year recovered for the first Time (95% CI)	Weighted proportion of patients with depression at 1-year recovered for the first Time (95% CI)
2y	1y	36	226	172	86	50.0(42.3-57.7)	52.3(44.9-59.8)
3y	2y	70	219	145	34	23.4(16.8-31.2)	23.4(16.5-30.3)
4y	3y	99	215	120	11	9.2(4.7-15.8)	7.6(2.8-12.3)
5y	4y	116	176	142	26	18.3(12.3-25.7)	17.6(11.4-23.9)
6y	5y	137	226	71	6	8.5(3.2-17.5)	6.7(0.9-12.5)
7y	6y	156	224	54	3	5.6(1.2-15.4)	5.5(1.1-13.1)
8y	7y	167	228	39	1	2.6(0.1-13.5)	2.6(0.1-9.8) [b]
9y	8y	187	215	32	3	8.9(1.9-23.7)	8.4(1.5-20.3) [b]
10y	9y	197	210	27	2	7.1(0.9-23.5)	5.3(0.2-16.6) [b]
11y	10y	204	203	27	2	7.1(0.9-23.5)	9.1(1.4-22.6) [b]
12y	11y	207	208	19	1	5.3(0.1-26.0)	2.2(0.6-13.3) [b]
13y	12y	212	207	15	0	0	0
14y	13y	213	211	10	0	0	0
15y	14y	216	196	22	0	0	0
16y	15y	217	195	22	0	0	0
17y	16y	218	204	12	0	0	0
18y	17y	221	206	7	0	0	0

Notes:
a: proportion of recovery calculated using IPW.
b: The logistic model to predict the probability of being completeness may be unstable since the number of complete cases was small. The estimate with IPW analysis should be read with caution.

Supplementary Table 14 Recurrence of depression after stroke (sensitivity analysis, exclude patients with pre-stroke depression)

A: Recurrent depression in patients with depression at 3-months and recovered at 1-year after stroke

Recurrent time (time since stroke)	Recurrent time (time since recovery)	Patients with incident depression at 3-months had assessment of recurrence at any time-point	Patients with incident depression at 3-months had depression recurred at any time-point	Cumulative recurrence rate of depression at each time-point	Weighted cumulative recurrence rate of depression at each time-point
2y	1y	100	34	34.0(24.8-44.2)	34.2(24.9-43.5)
3y	2y	114	54	47.4(37.9-56.9)	47.2(38.1-56.4)
4y	3y	118	71	60.2(50.7-69.1)	60.2(51.4-69.0)
5y	4y	130	78	60.0(51.0-68.5)	59.8(51.4-68.3)
6y	5y	130	80	61.5(52.6-69.9)	61.4(53.0-69.8)
7y	6y	130	80	61.5(52.6-69.9)	61.4(53.0-69.8)
8y	7y	130	81	62.3(53.4-70.7)	62.2(53.8-70.5)
9y	8y	130	84	64.6(55.8-72.8)	64.5(56.3-72.7)
10y	9y	130	85	65.4(56.5-73.5)	65.3(57.1-73.4)
11y	10y	130	85	65.4(56.5-73.5)	65.3(57.1-73.4)
12y	11y	130	85	65.4(56.5-73.5)	65.3(57.1-73.4)
13y	12y	130	85	65.4(56.5-73.5)	65.3(57.1-73.4)
14y	13y	130	85	65.4(56.5-73.5)	65.3(57.1-73.4)
15y	14y	130	85	65.4(56.5-73.5)	65.3(57.1-73.4)
16y	15y	130	85	65.4(56.5-73.5)	65.3(57.1-73.4)
17y	16y	130	85	65.4(56.5-73.5)	65.3(57.1-73.4)
18y	17y	130	85	65.4(56.5-73.5)	65.3(57.1-73.4)

B: Recurrent depression in patients with incident PSD at 1-year and recovered at 2-years after stroke

Recurrent time (time since stroke)	Recurrent time (time since recovery)	Patients with incident depression at 1-year had assessment of recurrence at any time-point	Patients with incident depression at 1-year had depression recurred at any time-point	Cumulative recurrence rate of depression at each time-point	Weighted Cumulative recurrence rate of depression at each time-point
3y	1y	57	21	36.8(24.4-50.7)	36.9(24.4-49.4)
4y	2y	64	32	50.0(37.2-62.8)	50.0(37.8-62.2)
5y	3y	72	44	61.1(48.9-72.4)	61.0(49.8-72.2)
6y	4y	72	45	62.5(50.3-73.6)	62.3(51.5-73.1)
7y	5y	72	45	62.5(50.3-73.6)	62.3(51.5-73.1)
8y	6y	72	46	63.9(51.7-74.9)	63.8(52.8-74.8)
9y	7y	73	48	65.8(53.7-76.5)	65.6(55.1-76.1)
10y	8y	73	49	67.1(55.1-77.7)	67.0(56.4-77.6)
11y	9y	73	49	67.1(55.1-77.7)	67.0(56.4-77.6)
12y	10y	73	49	67.1(55.1-77.7)	67.0(56.4-77.6)
13y	11y	73	49	67.1(55.1-77.7)	67.0(56.4-77.6)
14y	12y	73	49	67.1(55.1-77.7)	67.0(56.4-77.6)
15y	13y	73	50	68.5(56.6-78.9)	68.4(57.8-79.0)
16y	14y	73	50	68.5(56.6-78.9)	68.4(57.8-79.0)
17y	15y	73	50	68.5(56.6-78.9)	68.4(57.8-79.0)
18y	16y	73	50	68.5(56.6-78.9)	68.4(57.8-79.0)

Supplementary Table 15 Prevalence of depression up to 18-years after stroke (sensitivity analysis, excluded patients took antidepressants)

Time Since Stroke	Number of patients assessed for depression at each time-point	Number of patients with depression at each time-point	Prevalence of depression at each time-point (95%CI)	Weighted Prevalence of depression at each time-point (95% CI) [a]	Number of patients with mild depression at each time-point	Prevalence of mild depression at each time-point (95%CI)	Number of patients with severe depression at each time-point	Prevalence of severe depression (at each time-point 95%CI)
3m[a]	1731	515	29.8(27.6-32.0)	27.3(26.2-28.4)	303	17.2(15.4-18.9)	231	13.0(11.5-14.7)
1y[a]	1798	492	27.4(25.3-29.5)	31.6(30.3-32.9)	164	16.0(14.3-17.7)	129	12.1(10.2-14.3)
2y[a]	1062	293	27.6(24.9-30.4)	28.6(27.3-29.8)	287	15.4(13.3-17.8)	205	11.4(10.0-13.0)
3y[a]	1205	363	30.1(27.5-32.8)	32.5(31.2-33.7)	211	17.5(15.4-19.8)	152	12.6(10.8-14.6)
4y[a]	995	285	28.6(25.9-31.6)	30.7(29.5-32.0)	144	14.5(12.3-16.8)	141	14.2(12.1-16.5)
5y[a]	1110	325	29.3(26.6-32.1)	32.2(30.9-33.5)	180	16.7(14.5-19.0)	145	13.1(11.1-15.2)
6y[a]	696	187	26.9(23.6-30.3)	30.9(29.6-32.2)	109	15.7(13.0-18.6)	78	11.1(9.0-13.8)
7y[a]	575	167	29.0(25.4-32.9)	30.7(29.4-32.0)	86	15.0(12.1-18.1)	81	14.1(11.3-17.2)
8y[a]	475	125	26.3(22.4-30.6)	30.9(29.5-32.3)	63	13.3(10.3-16.6)	62	13.1(10.2-16.4)
9y	367	109	29.7(25.1-34.7)	36.7(35.1-38.4)	63	17.2(13.5-21.4)	46	12.5(9.3-16.4)
10y	295	90	30.6(25.3-36.1)	30.6(28.9-32.2)	45	15.3(11.3-19.9)	45	15.3(11.3-19.9)
11y	244	66	27.0(21.6-33.1)	31.8(30.0-33.7)	38	15.6(11.3-20.7)	28	11.5(7.8-16.2)
12y	179	60	33.5(26.7-40.9)	31.0(28.9-33.3)	30	16.8(11.6-23.1)	30	16.8(11.6-23.1)
13y	131	38	29.0(21.4-37.6)	31.6(29.3-34.1)	16	12.2(7.1-19.1)	22	16.8(10.8-24.3)
14y	109	38	34.9(26.0-44.6)	35.8(33.8-37.8)	19	17.4(10.8-25.9)	19	17.4(10.8-25.9)
15y	200	68	34.0(27.5-41.0)	37.3(35.2-39.4)	39	19.5(14.2-25.7)	29	14.5(9.9-20.2)
16y	165	55	33.3(26.2-41.1)	35.4(33.2-37.7)	29	17.6(12.1-24.3)	26	15.8(10.6-22.2)
17y	133	42	31.6(23.8-40.2)	36.8(27.6-46.7)	27	20.3(13.8-28.1)	15	18.8(12.5-26.5)
18y	106	39	36.8(27.6-46.7)	37.6(35.2-40.2)	23	21.7(14.3-30.8)	16	15.1(8.9-23.4)

Note:

a: The differences in the prevalence of PSD between this subgroup (patients without antidepressants) and full sample was statistically significant ($p<0.05$).

Supplementary Table 16 Prevalence of depression up to 18 years after stroke in patients took antidepressants

Time since stroke	Number of patients assessed at each time-point	Number of patients with depression at each time-point	Prevalence of depression at each time-point (95%CI)	Number of patients with mild depression at each time-point	Prevalence of mild depression at each time-point (95%CI)	Number of patients with severe depression at each time-point	Prevalence of severe depression (at each time-point 95%CI)
3m	453	235	51.9(47.2-56.6)	85	18.8(15.3-22.7)	150	33.1(28.8-37.7)
1y	497	250	50.3(45.8-54.8)	101	20.3(16.9-24.1)	149	30.0(26.0-34.2)
2y	320	140	43.8(38.2-49.4)	60	18.8(14.6-23.5)	80	25.0(20.4-30.1)
3y	337	156	46.3(40.9-51.8)	70	20.8(16.6-25.5)	86	25.5(20.9-30.5)
4y	310	161	51.9(46.2-57.6)	69	22.3(17.8-27.3)	92	29.7(24.6-35.1)
5y	340	177	52.1(46.6-57.5)	84	24.7(20.2-29.6)	93	27.4(22.7-32.4)
6y	235	110	46.8(40.3-53.4)	45	19.1(14.3-24.8)	65	27.7(22.0-33.9)
7y	199	104	52.3(45.1-59.4)	45	22.6(17.0-29.1)	59	29.6(23.4-36.5)
8y	167	89	53.3(45.4-61.0)	38	22.8(16.6-29.9)	51	30.5(23.7-38.1)
9y	129	69	53.5(44.5-62.3)	36	27.9(20.4-36.6)	33	25.6(18.3-34.0)
10y	102	50	49.5(39.4-59.6)	19	18.8(11.7-27.8)	31	30.7(21.9-40.7)
11y	85	51	60.0(48.8-70.5)	22	25.9(17.0-36.5)	29	34.1(24.2-45.2)
12y	72	33	45.8(34.0-58.0)	12	16.7(8.9-27.3)	21	29.2(19.0-41.1)
13y	57	28	49.1(35.6-62.7)	10	17.5(8.7-29.9)	18	31.6(19.9-45.2)
14y	44	25	56.8(41.0-71.7)	11	25.0(13.2-40.3)	14	31.8(18.6-47.6)
15y	82	43	52.4(41.1-63.6)	18	22.0(13.6-32.5)	25	30.5(20.8-41.6)
16y	67	39	58.2(45.5-70.2)	17	25.4(15.5-37.5)	22	32.8(21.8-45.4)
17y	53	28	52.8(38.6-66.7)	13	24.5(13.8-38.3)	15	28.3(16.8-42.3)
18y	39	27	69.2(52.4-83.0)	10	25.6(13.0-42.1)	17	43.6(27.8-60.4)

Supplementary Table 17 Recovery in patients with depression after stroke (sensitivity analysis, exclude patients with antidepressants)

A: Recovery in patients with depression at 3-months after stroke

Recovery Time (time since stroke)	Recovery Time (time since depression)	Patients with mild depression at 3-months died at each time-point	Patients with mild depression at 3-months lost to follow-up at each time-point	Patients with mild depression at 3-months with complete follow-up	Patients with mild depression at 3-months recovered for the first time	Proportion of patients with depression at 3-months recovered for the first time (95% CI)	Weighted proportion of patients with depression at 3- months recovered for the first time (95% CI) [a]
1y	1y	47	173	314	167	53.2(47.5-58.8)	53.3(48.9-57.7)
2y	2y	83	280	171	34	13.5(14.2-26.7)	18.5(15.2-22.1)
3y	3y	127	251	156	21	13.5(8.5-19.8)	13.4(10.6-16.6)
4y	4y	152	258	124	12	9.7(5.1-16.3)	11.3(8.7-14.4)
5y	5y	179	217	138	21	15.2(9.7-22.3)	9.6(7.3-12.5)
6y	6y	200	259	75	5	6.7(2.2-14.9)	11.1(8.5-14.2)
7y	7y	219	249	66	3	4.5(0.9-12.7)	3.8(2.3-5.8)
8y	8y	231	251	52	1	1.9(0.1-10.3)	1.0(0.3-2.6)
9y	9y	240	259	35	0	0	0
10y	10y	253	248	33	0	0	0
11y	11y	264	241	29	0	0	0
12y	12y	271	246	17	0	0	0
13y	13y	273	247	14	0	0	0
14y	14y	276	248	10	0	0	0
15y	15y	280	235	19	0	0	0
16y	16y	281	232	21	0	0	0
17y	17y	281	237	16	0	0	0
18y	18y	282	239	13	0	0	0

Notes:
a: proportion of recovery calculated using IPW.
b: The logistic model to predict the probability of being completeness may be unstable since the number of complete cases was small. The estimate with IPW analysis should be read with caution.

B: Recovery in patients with incident PSD at 1-year after stroke

Recovery time (time since stroke)	Recovery time (time since depression)	Patients with depression at 1-year died at each time-point	Patients with depression at 1-year lost to follow-up at each time-point	Patients with depression at 1-year with complete follow-up	Patients with depression at 1-year recovered for the first time	Proportion of patients with depression at 1-year recovered for the first Time (95% CI)	Weighted proportion of patients with depression at 1-year recovered for the first Time (95% CI)
2y	1y	35	193	117	61	52.1(42.7-61.5)	56.8(51.4-62.2)
3y	2y	66	187	92	21	22.8(14.7-32.8)	24.1(19.6-29.0)
4y	3y	90	173	82	10	12.2(6.0-21.3)	14.3(10.8-18.5)
5y	4y	107	139	99	17	17.2(10.3-26.1)	16.9(12.9-21.4)
6y	5y	123	179	43	3	7.0(1.5-19.1)	6.3(3.9-9.5)
7y	6y	133	179	33	3	9.1(1.9-24.3)	5.6(3.3-8.8)[b]
8y	7y	144	179	22	1	4.5(0.1-22.8)	2.8(0.9-6.4)[b]
9y	8y	159	165	21	3	9.5(1.2-30.4)	21.3(14.5-29.4)[b]
10y	9y	164	162	19	2	10.5(1.3-37.1)	19.5(13.2-27.3)[b]
11y	10y	168	158	19	2	5.3(0.1-26.0)	1.6(0.2-5.6)[b]
12y	11y	170	159	16	1	6.3(0.2-30.2)	5.3(2.0-11.2)[b]
13y	12y	174	160	11	0	0	0
14y	13y	175	164	6	0	0	0
15y	14y	179	153	13	0	0	0
16y	15y	180	151	14	0	0	0
17y	16y	180	159	6	0	0	0
18y	17y	182	159	4	0	0	0

Notes:
a: proportion of recovery calculated using IPW.
b: The logistic model to predict the probability of being completeness may be unstable since the number of complete cases was small. The estimate with IPW analysis should be read with caution.

Supplementary Table 18 Recurrence of depression after stroke (sensitivity analysis, exclude patients took antidepressants)

A: Recurrent depression in patients with depression at 3-months and recovered at 1-year after stroke

Recurrent time (time since stroke)	Recurrent time (time since recovery)	Patients with incident depression at 3-months had assessment of recurrence at any time-point	Patients with incident depression at 3-months had depression recurred at any time-point	Cumulative recurrence rate of depression at each time-point	Weighted cumulative recurrence rate of depression at each time-point
2y	1y	78	26	33.3(23.1-44.9)	26.2(19.7-33.6)
3y	2y	94	46	48.9(38.5-59.5)	44.7(36.8-52.7)
4y	3y	95	54	56.8(46.3-67.0)	59.7(51.7-67.4)
5y	4y	103	60	58.3(48.1-67.9)	59.2(51.1-66.8)
6y	5y	103	61	59.2(49.1-68.8)	61.0(53.0-68.6)
7y	6y	103	61	59.2(49.1-68.8)	61.0(53.0-68.6)
8y	7y	103	62	60.2(52.0-71.5)	61.6(53.6-69.2)
9y	8y	103	64	62.1(52.0-71.5)	62.9(54.9-70.4)
10y	9y	103	65	63.1(53.0-72.4)	63.5(55.5-71.0)
11y	10y	103	65	63.1(53.0-72.4)	63.5(55.5-71.0)
12y	11y	103	65	63.1(53.0-72.4)	63.5(55.5-71.0)
13y	12y	103	65	63.1(53.0-72.4)	63.5(55.5-71.0)
14y	13y	103	65	63.1(53.0-72.4)	63.5(55.5-71.0)
15y	14y	103	65	63.1(53.0-72.4)	63.5(55.5-71.0)
16y	15y	103	65	63.1(53.0-72.4)	63.5(55.5-71.0)
17y	16y	103	65	63.1(53.0-72.4)	63.5(55.5-71.0)
18y	17y	103	65	63.1(53.0-72.4)	63.5(55.5-71.0)

B: Recurrent depression in patients with incident PSD at 1-year and recovered at 2-years after stroke

Recurrent time (time since stroke)	Recurrent time (time since recovery)	Patients with incident depression at 1-year had assessment of recurrence at any time-point	Patients with incident depression at 1-year had depression recurred at any time-point	Cumulative recurrence rate of depression at each time-point	Weighted Cumulative recurrence rate of depression at each time-point
3y	1y	39	12	30.8(17.0-47.6)	29.3(18.1-42.7)
4y	2y	44	19	43.2(28.3-59.0)	37.5(24.9-51.5)
5y	3y	47	26	55.3(40.1-69.8)	58.1(42.1-73.0)
6y	4y	47	27	57.4(42.2-71.7)	60.5(44.4-75.0)
7y	5y	47	27	57.4(42.2-71.7)	60.5(44.4-75.0)
8y	6y	47	28	59.6(44.3-73.6)	62.8(46.7-77.0)
9y	7y	47	28	59.6(44.3-73.6)	62.8(46.7-77.0)
10y	8y	47	28	59.6(44.3-73.6)	62.8(46.7-77.0)
11y	9y	47	28	59.6(44.3-73.6)	62.8(46.7-77.0)
12y	10y	47	28	59.6(44.3-73.6)	62.8(46.7-77.0)
13y	11y	47	28	59.6(44.3-73.6)	62.8(46.7-77.0)
14y	12y	47	28	59.6(44.3-73.6)	62.8(46.7-77.0)
15y	13y	47	28	59.6(44.3-73.6)	62.8(46.7-77.0)
16y	14y	47	29	61.7(46.4-75.5)	65.1(49.1-79.0)
17y	15y	47	29	61.7(46.4-75.5)	65.1(49.1-79.0)
18y	16y	47	29	61.7(46.4-75.5)	65.1(49.1-79.0)

Supplementary Figure 1. Number of participants included in the analysis at each follow up time-point

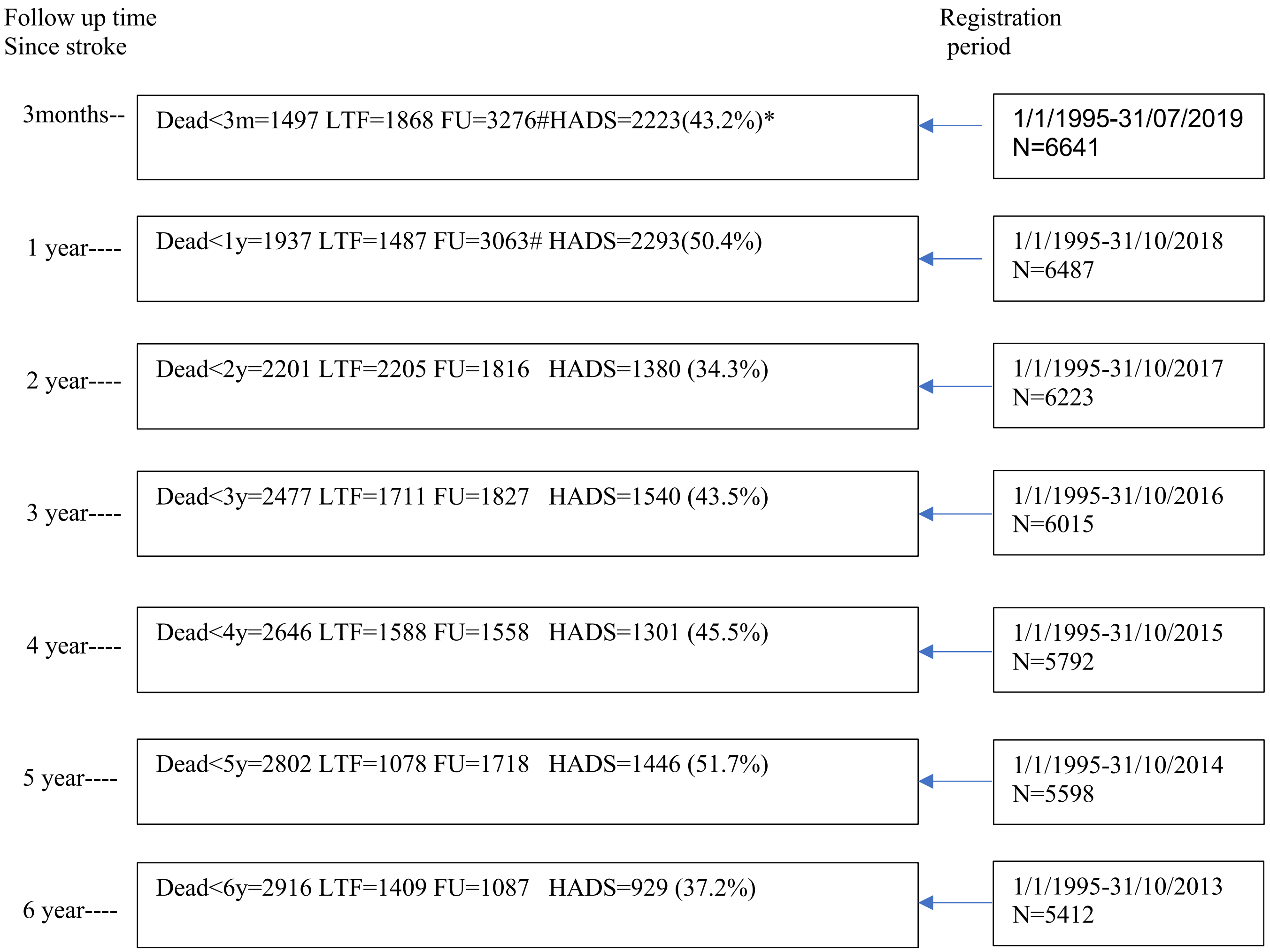

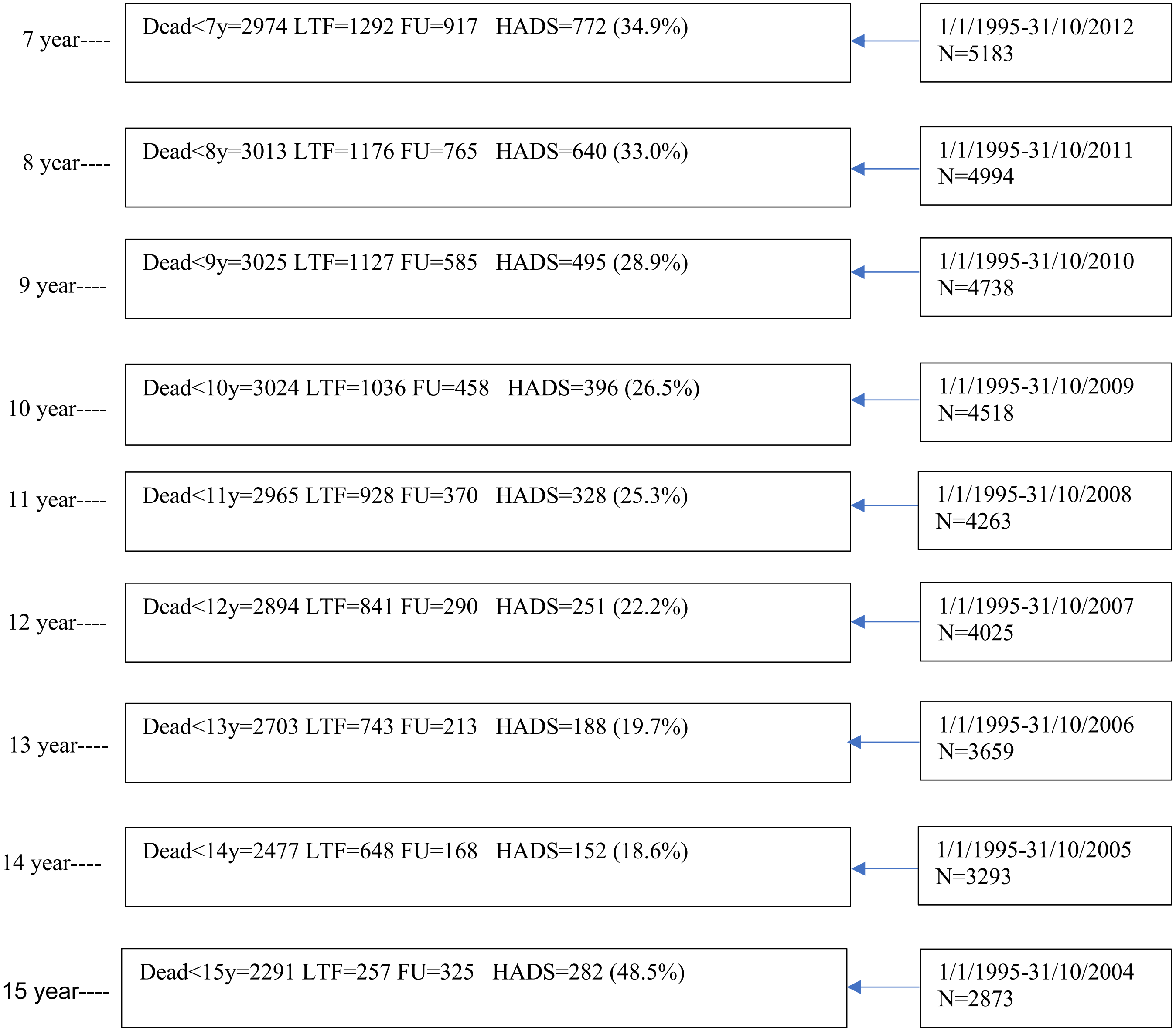
7 year----
Dead<7y=2974 LTF=1292 FU=917 HADS=772 (34.9%)
1/1/1995-31/10/2012
N=5183
8 year----
Dead<8y=3013 LTF=1176 FU=765 HADS=640 (33.0%)
1/1/1995-31/10/2011
N=4994
9 year----
Dead<9y=3025 LTF=1127 FU=585 HADS=495 (28.9%)
1/1/1995-31/10/2010
N=4738
10 year----
Dead<10y=3024 LTF=1036 FU=458 HADS=396 (26.5%)
1/1/1995-31/10/2009
N=4518
11 year----
Dead<11y=2965 LTF=928 FU=370 HADS=328 (25.3%)
1/1/1995-31/10/2008
N=4263
12 year----
Dead<12y=2894 LTF=841 FU=290 HADS=251 (22.2%)
1/1/1995-31/10/2007
N=4025
13 year----
Dead<13y=2703 LTF=743 FU=213 HADS=188 (19.7%)
1/1/1995-31/10/2006
N=3659
14 year----
Dead<14y=2477 LTF=648 FU=168 HADS=152 (18.6%)
1/1/1995-31/10/2005
N=3293
15 year----
Dead<15y=2291 LTF=257 FU=325 HADS=282 (48.5%)
1/1/1995-31/10/2004
N=2873

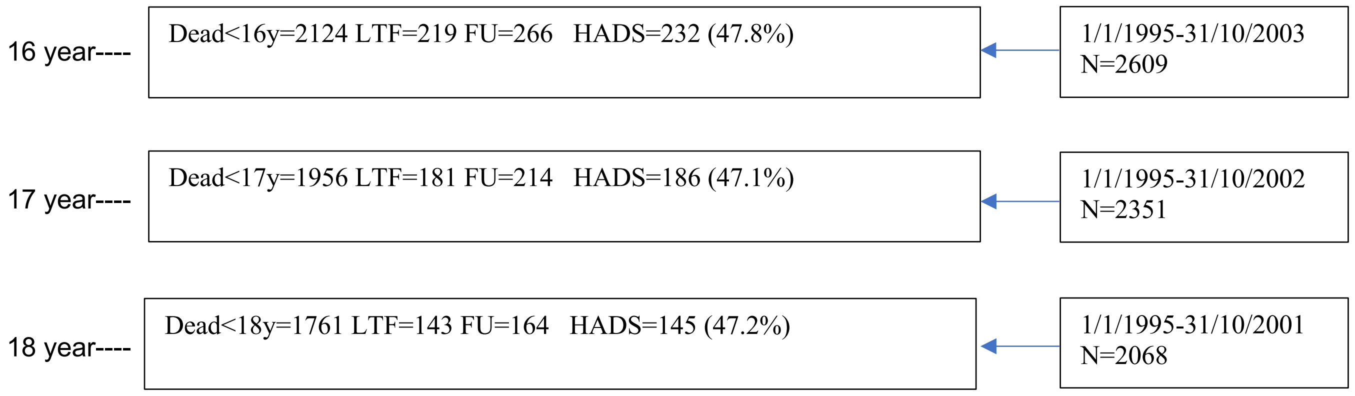

Notes:
Some patients who were followed up could not be assessed with HADS due to cognitive or communication impairment.
N= number of patients registered.
HADS=number of patients completing the depression scale.
FU= Number of patients followed up at each time point.
LTF=Number of patients lost to follow up at each time point.
#: Patients registered in 1995 and 1996 were not assessed for depression at this point as HADs was routinely collected from 1997.
*: Proportion of patients assessed with HADS over the total patients followed up and lost to follow up

Supplementary Figure 2 Number of participants included in the sensitivity analysis at each follow up time-point (Sensitivity analysis, included patients recruited between Jan 1995 and Mar 2010)

Follow up time Since stroke		Registration period
3months---	Dead<3m=1187 LTF=1258 FU=2162# HADS=1285(37.6%)*	1/1/1995-31/3/2010 N=4608
1 year----	Dead<1y=1545 LTF=901 FU=2162# HADS=1508(49.2%)	1/1/1995-31/3/2010 N=4608
2 year----	Dead<2y=1796 LTF=1186 FU=1626 HADS=1217(43.3%)	1/1/1995-31/3/2010 N=4608
3 year----	Dead<3y=2047 LTF=806 FU=1755 HADS=1482(57.9%)	1/1/1995-31/3/2010 N=4608
4 year----	Dead<4y=2243 LTF=802 FU=1563 HADS=1305(55.2%)	1/1/1995-31/3/2010 N=4608
5 year----	Dead<5y=2420 LTF=864 FU=1324 HADS=1101(50.3%)	1/1/1995-31/3/2010 N=4608
6 year----	Dead<6y=2350 LTF=695 FU=1086 HADS=928(52.1%)	1/1/1995-31/3/2008 N=4131

7 year----	Dead<7y=2303 LTF=583 FU=917 HADS=772(51.5%)	1/1/1995-31/3/2007 N=3803
8 year----	Dead<8y=2198 LTF=476 FU=764 HADS=639(51.5%)	1/1/1995-31/3/2006 N=3438
9 year----	Dead<9y=2068 LTF=388 FU=583 HADS=494(50.9%)	1/1/1995-31/3/2005 N=3039
10 year----	Dead<10y=1945 LTF=310 FU=457 HADS=395(51.5%)	1/1/1995-31/3/2004 N=2712
11 year----	Dead<11y=1839 LTF=258 FU=369 HADS=328(52.3%)	1/1/1995-31/3/2003 N=2466
12 year----	Dead<12y=1680 LTF=211 FU=289 HADS=250(50.0%)	1/1/1995-31/3/2002 N=2180
13 year----	Dead<13y=1500 LTF=184 FU=213 HADS=188(47.4%)	1/1/1995-31/3/2001 N=1897
14 year----	Dead<14y=1338 LTF=134 FU=167 HADS=151(51.0%)	1/1/1995-31/3/2000 N=1639
15 year----	Dead<15y=1313 LTF=112 FU=154 HADS=137(51.5%)	1/1/1995-31/12/1999 N=1579

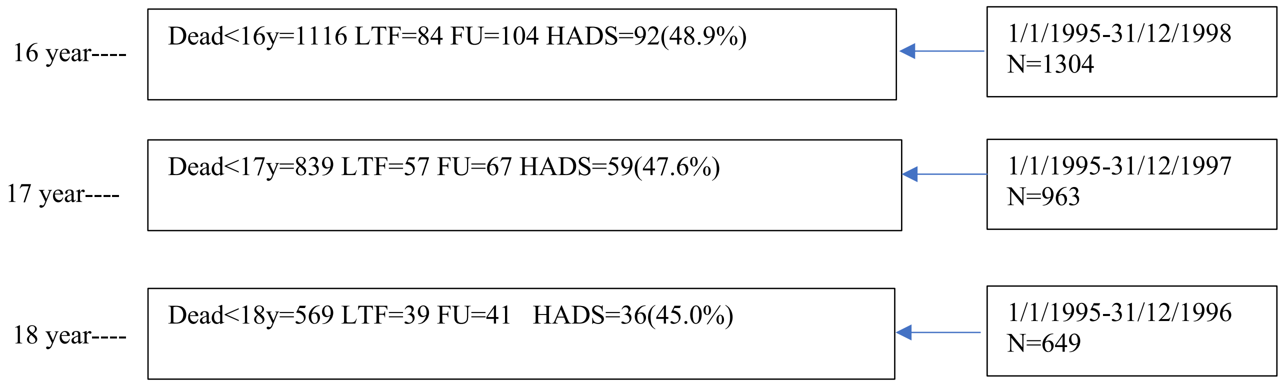

Notes:
Some patients who were followed up could not be assessed with HADS due to cognitive or communication impairment.
N= number of patients registered.
HADS=number of patients completing the depression scale.
FU= Number of patients followed up at each time point.
LTF=Number of patients lost to follow up at each time point.
#: Patients registered in 1995 and 1996 were not assessed for depression at this point as HADs was routinely collected from 1997.
*: Proportion of patients assessed with HADS over the total patients followed up and lost to follow up

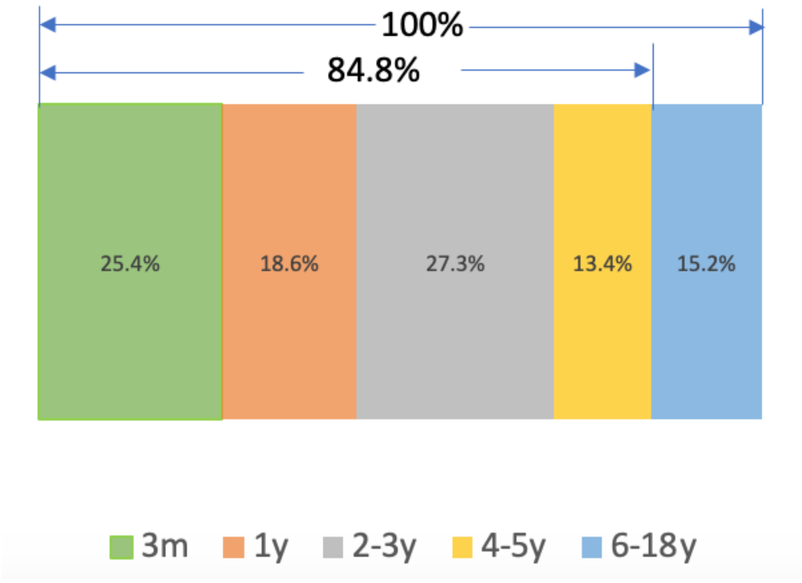

Supplementary Figure 3 Time since stroke of incident depression in patients recruited Jan 1995-Mar 2010

Note: Among all the incident depression in patients recruited Jan 1995-Mar 2010 during the 18-years follow-up, 25.4% (95%CI: 23.3-27.6) occurred within 3-months and 84.8% (82.9-86.5) occurred within 5-years after stroke.

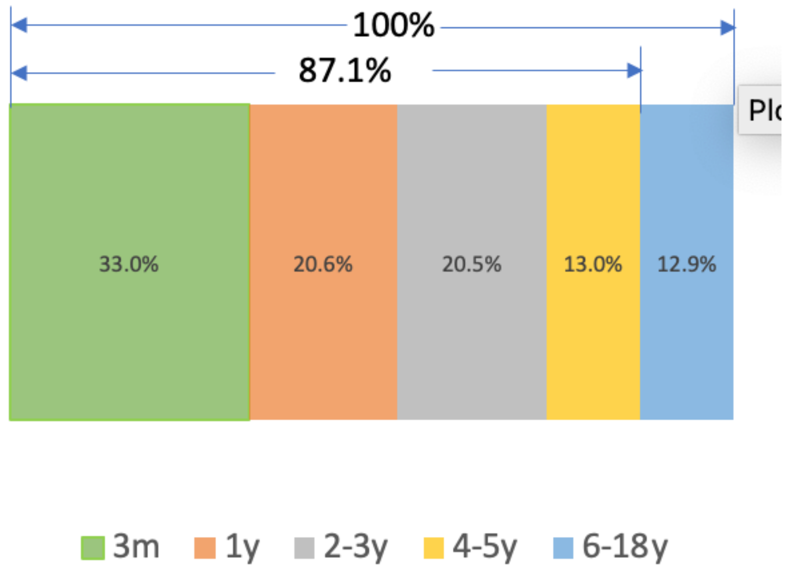

Supplementary Figure 4 Time since stroke of incident depression in patients without pre-stroke depression

Note: Among all the incident depression in patients without pre-stroke depression during the 18-years follow-up, 33.0% (95%CI: 31.0-35.1) occurred within 3-months and 87.1% (85.6-88.5) occurred within 5-years after stroke.

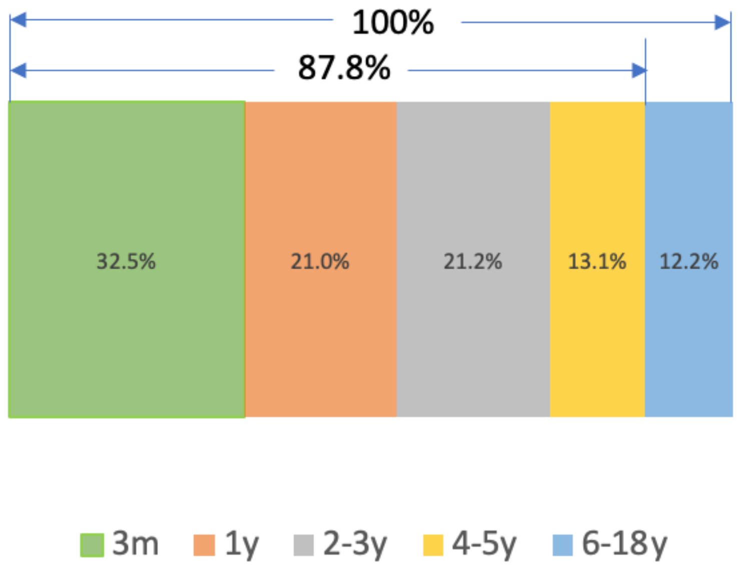

Supplementary Figure 5 Time since stroke of incident depression in patients did not take antidepressants

Note: Among all the incident depression in patients without pre-stroke depression during the 18-years follow-up, 32.5% (95%CI: 30.2-34.8) occurred within 3-months and 87.8% (86.2-89.4) occurred within 5-years after stroke.
